# Genomic study and lipidomic bioassay of *Leeuwenhoekiella parthenopeia*: A novel rare biosphere marine bacterium that inhibits tumor cell viability

**DOI:** 10.3389/fmicb.2022.1090197

**Published:** 2023-01-06

**Authors:** Giuliano Gattoni, Rafael R. de la Haba, Jesús Martín, Fernando Reyes, Cristina Sánchez-Porro, Antonia Feola, Candida Zuchegna, Shaday Guerrero-Flores, Mario Varcamonti, Ezio Ricca, Nelly Selem-Mojica, Antonio Ventosa, Paulina Corral

**Affiliations:** ^1^Department of Biology, University of Naples Federico II, Naples, Italy; ^2^Department of Microbiology and Parasitology, Faculty of Pharmacy, University of Sevilla, Sevilla, Spain; ^3^Fundación MEDINA, Granada, Spain; ^4^Centro de Ciencias Matemáticas, Universidad Nacional Autónoma de México (UNAM), Morelia, Mexico

**Keywords:** *Leeuwenhoekiella*, rare biosphere, diel vertical migration (DVM), biosynthetic profiling, membrane lipids, antiproliferative, tumor cells

## Abstract

The fraction of low-abundance microbiota in the marine environment is a promising target for discovering new bioactive molecules with pharmaceutical applications. Phenomena in the ocean such as diel vertical migration (DVM) and seasonal dynamic events influence the pattern of diversity of marine bacteria, conditioning the probability of isolation of uncultured bacteria. In this study, we report a new marine bacterium belonging to the rare biosphere, *Leeuwenhoekiella parthenopeia* sp. nov. Mr9^T^, which was isolated employing seasonal and diel sampling approaches. Its complete characterization, ecology, biosynthetic gene profiling of the whole genus *Leeuwenhoekiella*, and bioactivity of its extract on human cells are reported. The phylogenomic and microbial diversity studies demonstrated that this bacterium is a new and rare species, barely representing 0.0029% of the bacterial community in Mediterranean Sea metagenomes. The biosynthetic profiling of species of the genus *Leeuwenhoekiella* showed nine functionally related gene cluster families (GCF), none were associated with pathways responsible to produce known compounds or registered patents, therefore revealing its potential to synthesize novel bioactive compounds. *In vitro* screenings of *L. parthenopeia* Mr9^T^ showed that the total lipid content (lipidome) of the cell membrane reduces the prostatic and brain tumor cell viability with a lower effect on normal cells. The lipidome consisted of sulfobacin A, WB 3559A, WB 3559B, docosenamide, topostin B-567, and unknown compounds. Therefore, the bioactivity could be attributed to any of these individual compounds or due to their synergistic effect. Beyond the rarity and biosynthetic potential of this bacterium, the importance and novelty of this study is the employment of sampling strategies based on ecological factors to reach the hidden microbiota, as well as the use of bacterial membrane constituents as potential novel therapeutics. Our findings open new perspectives on cultivation and the relationship between bacterial biological membrane components and their bioactivity in eukaryotic cells, encouraging similar studies in other members of the rare biosphere.

## 1. Introduction

The exploration of the uncultivated marine microbiota is a promising target in the search for alternative drugs to meet the urgent therapeutic needs where the current pharmacotherapy is ineffective (García-Davis et al., 2021; Zhou et al., 2021). Every year, new cases of cancer are registered worldwide, and many of them are resistant forms that do not respond to available treatments (Maeda and Khatami, 2018). This low therapeutic efficacy reduces life expectancy, as cancer is among the main leading causes of death (Siegel et al., 2021). In this scenario, the isolation of uncultured bacteria is a current trend, in addition to culture-independent studies, since there is a need to explore the true metabolic and intrinsic characteristics both in their habitat and *in vitro* (Pascoal et al., 2020; Sanz-Sáez et al., 2020). Most of the discovered bioactive compounds were derived from only 1% of the total microbial diversity known so far (Baker et al., 2021). An outstanding example is a salinosporamide A, an agent in phase III clinical trial for the treatment of glioblastoma produced by the rare marine actinobacteria of the genus *Salinispora* (Fenical et al., 2009; Roth et al., 2020; Boccellato et al., 2021). Despite this successful precedent, the huge biosynthetic potential remains in the great prokaryotic diversity not yet cultivated as predicted by microbiome studies (Paoli et al., 2022). The low rate of cultivation of new taxa is even more challenging when targeting the very low-abundant fraction (0.1 to 0.01% of relative abundance) known as the rare biosphere (Sogin et al., 2006; Pedrós-Alió, 2007). To overcome this limitation, prokaryotic diversity is being addressed by considering environmental variabilities that affect the microbial population distribution, such as biomass migration and seasonality (Shade et al., 2014; Cram et al., 2015; Mestre et al., 2020).

With the aim to cultivate novel and rare bacteria with anticancer potential, here, we employed sampling strategies considering seasonality and natural dynamic processes in the marine environment rather than culture techniques *per se*. The largest biomass dynamic event in the ocean is the diel vertical migration (DVM) where a part of the inhabitants, especially zooplankton, ascends from the depths to the surface waters during the night or light absence and moves downward during the day (Bosch and Rowland Taylor, 1973; Omand et al., 2021). In the same way, seasonality influences the pattern of microbial diversity as the water column in the ocean stratifies during the warm period and mixes during the cold months (Giovannoni and Vergin, 2012; Haro-Moreno et al., 2018; Mena et al., 2020). In this study, cold seasons and night were identified as the best sampling time since the water column is mixed and DVM brings planktonic particulate as drivers of microbial transfer from the depths during the night (Gilbert et al., 2012; Fuhrman et al., 2015; Kelly et al., 2019). In both cases, the microbial community is redistributed, increasing the possibility of reaching uncommon bacteria. Furthermore, it has been demonstrated that surface and deep-sea prokaryotic communities are strongly connected in the oceans through the transport of sinking particles (Mestre et al., 2018; Wenley et al., 2021). By employing this approach, a novel and rare marine bacterium, that we have designated as *Leeuwenhoekiella parthenopeia* strain Mr9^T^, was isolated from reef seawater in the Gulf of Naples, Italy, from night sampling series during the late autumn. Herein, we fully characterized this strain and inferred its specialized metabolism through the genome by predicting unknown biosynthetic gene clusters (BGCs) of pharmacological relevance (Agrawal et al., 2017; Belknap et al., 2020; Scherlach and Hertweck, 2021). Similarly, the biosynthetic potential of all described species of the genus *Leeuwenhoekiella* was genomically assessed by determining the functional homologies based on gene cluster families (GCFs) (Navarro-Muñoz et al., 2020). Considering that most BGCs of the secondary metabolism are not expressed *in vitro* or are not released outside the cell (Letzel et al., 2017), such as in the case of *L. parthenopeia* Mr9^T^, we investigated the structural constituents of the bacterial membrane, specifically the lipidome. The bacterial lipid composition variates to adapt in response to environmental changes maintaining the optimal functional properties of the membrane (Angelini et al., 2012; Chwastek et al., 2020). The lipid profile is used as a chemotaxonomic marker in the characterization of bacteria and archaea and can be different among species of the same genus (de la Haba et al., 2018), but there are no studies on bacterial membrane lipid composition and its effect on eukaryotic cells as such. Our study demonstrates that the total lipid extract (TLE) from *L. parthenopeia* Mr9^T^ affects tumor cell viability of prostate adenocarcinoma DU-145, and glioblastoma U-87 MG cell lines with a lower effect on the normal cell line HaCaT. The genus *Leeuwenhoekiella*, the object of this study, includes chemoorganotrophic Gram-negative bacteria, producing non-diffusible yellow-pigmented colonies with gliding motility (Nedashkovskaya et al., 2005; Tahon et al., 2020). Currently, this genus includes only seven species (Parte et al., 2020) derived from marine or sea-related habitats, and no bioactive compounds that impair tumor cell viability produced by strains of these species have been reported to date.

## 2. Materials and methods

### 2.1. Sampling and isolation

Serial weekly nightly samplings were carried out in the reef of the Gulf of Naples, Italy 40°49′48.9″N 14°13′31.8″E, during the fall and winter of 2020–2021. The selection of diel and cold seasons was based on previous studies (Gilbert et al., 2012; Fuhrman et al., 2015; Kelly et al., 2019) that demonstrate that the water column is mixed during these periods and DVM brings planktonic particulate acting as drivers of microbial transfer from the depths during the night, thus suggesting fall, winter, and night as the best sampling time. The salinity, pH, conductivity, particulate (total dissolved solids, TDS), and temperature were measured *in situ* with a refractometer and a digital multiparameter pH/EC/TDS/TEMP, Hanna HI-9812-51.

Five liters of seawater were collected from shallow waters (∼1 m) each time and homogenized by magnetic stirring. Aliquots of 40 mL of each sample were vortexed for 10 min, and 1 mL was serially diluted 10-fold in 9 mL of sterile seawater to a 10^–6^ dilution. A total of 100 μL of each dilution was inoculated on four different solid minimal media designed to cultivate oligotrophic and heterotrophic bacteria, formulated with filter-sterilized natural seawater as the base component and varying the mineral composition. The plates were incubated on the laboratory bench to follow the light day cycle at room temperature (∼21°C) and aerobic conditions. The growth was checked daily, and the smallest colonies were picked and streaked on the same medium until obtaining axenic cultures.

The strain Mr9^T^ was isolated from the night sampling series collected in November 2020 at 1-m depth after 4 days of incubation in seawater oligotrophic medium (SWOM) with the following composition (g/L of natural seawater): casamino acids, 0.5; yeast extract, 1.0; tryptone, 1.0; and agar, 15.0. This medium was supplemented with glycerol, 0.1 M; mineral solution, 500 μL/L; and vitamin solution, 500 μL/L. All supplement solutions were previously sterilized by filtration and added after autoclaving.

The mineral solution compositions are as follows (mg/L): *Na-EDTA, 250.0; ZnSO_4_ 7H_2_O, 1095.0; FeSO_4_ 7 H_2_O, 500; MnSO_4_ H_2_O, 154; CuSO_4_ 5H_2_O, 39.2; Co(NO_3_)_2_ 6H_2_O, 24.8; Na_2_B_4_O_7_ 10H_2_O, 17.7. Distilled water up to 1,000 mL. *Dissolve the EDTA and add a few drops of concentrated H_2_SO_4_ to retard the precipitation of the heavy metal ions.

Vitamin solution composition (mg/L): Biotin, 4.0; folic acid, 4.0; pyridoxine-HCl, 20.0; riboflavin, 10.0; thiamine-HCl 2H_2_O, 10.0; nicotinamide, 10.0; D-Ca-pantothenate, 10.0; vitamin B_12_, 0.20. Distilled water up to 1,000 mL. Stored in dark at 4°C.

After primary isolation and purification in SWOM, no growth was observed in synthetic medium marine agar (MA) 2260 (Difco), but the strain Mr9^T^ was adapted to grow on MA medium by adding the same supplement solutions as those used for SWOM, where a poor growth was visible after 10 days of incubation. After successive cultivation, the strain Mr9^T^ was well adapted to routine growth in MA without supplemental solutions, establishing the optimal growing condition to 4 days of incubation at 25°C. For long-term conservation, strain Mr9^T^ was cultivated in marine broth (MB) for 72 h and stored at −80°C in cryovials with 40% (v/v) of glycerol.

### 2.2. Characterization of new taxa

#### 2.2.1. Strain identification by amplification and sequencing of 16S rRNA gene

For the identification of strain Mr9^T^, the 16S rRNA gene was amplified using universal primers: 27F (5′-AGAG TTTGATCCTGGCTCAG-3′) and 1492R (5′-GGTTACCTT GTTACGACTT-3′). The PCR amplification was performed using a single colony of a 48-h culture of Mr9^T^ and resuspending it in 40 μL of a master mix as follows: *Reaction buffer 5×, 10 μL; primer forward 27F (20 μM), 1 μL; primer reverse 1492R (20 μM), 1 μL; *Taq* DNA polymerase MyTaq (Bioline), 1 μL; and H_2_O nuclease-free, 27 μL. *Reaction buffer 5× contains dNTPs, MgCl_2_, and enhancers. The reaction was performed using a thermal cycler T100 (BioRad, CA, USA) with the following settings: Initial denaturation for 5′ at 95°C, 25 cycles of 30″ at 94°C, 30″ at 50°C, 90″ at 72°C, and final extension for 10′ at 72°C.

The amplification was verified by 0.8% agarose gel electrophoresis in TBE and stained with SYBR Safe DNA Gel Stain 10,000× in dimethyl sulfoxide (DMSO) (Invitrogen, MA, USA), and the size was checked using the 1 Kb Plus DNA Ladder (Invitrogen, MA, USA).

The PCR product was purified using a silica-based membrane GeneJET PCR purification kit (Invitrogen, MA, USA) following the manufacturer’s protocol and sequenced by Stab Vida (Portugal) using the Sanger method. Sequence quality analysis and trimming were accomplished using the software 4Peaks V1.8 and contig assembly of the whole gene with ChromasPro [Research Resource Identifiers (RRIDs)]: (RRID: SCR_000229) v.2.1.10.

Sequence similarity search was performed against the EzBioCloud database (Yoon et al., 2017) with the 16S-based ID application, and with the NCBI BLAST (RRID: SCR_004870) against NCBI nr/nt Nucleotide (RRID: SCR_004860) database restricted to type-sequenced taxa and then also including uncultured/environmental sample sequences.

#### 2.2.2. Genome sequencing, assembly, annotation, and relatedness indexes

Genomic DNA was extracted using the DNeasy UltraClean kit (QIAGEN, Hilden, Germany) following the protocol for Gram-negative bacteria. DNA purity and integrity were checked by agarose gel electrophoresis (0.8%) and using NanoDrop 2000 Spectrophotometer (Thermo Scientific, MA, USA). The DNA was quantified employing the Qubit assay kit for double-stranded DNA broad range (dsDNA BR, Invitrogen) and measured using the Qubit 4 Fluorometer (Invitrogen, MA, USA).

The genome of strain Mr9^T^ was sequenced using Illumina NovaSeq 6000 platform (2 × 150 bp paired-end sequencing reads) at Novogene (Genomic sequencing center, Cambridge, UK). Quality control of raw reads was checked with FastQC (RRID: SCR_014583) v.0.11.9 and reads containing adapters and low-quality bases (*Q* ≤ 20) were removed with Trimmomatic (RRID: SCR_011848) v.0.36 (Bolger et al., 2014). *De novo* assembly of the reads was performed using SPAdes (RRID: SCR_000131) v.3.15.4 (Bankevich et al., 2012) and SOAP3 (RRID: SCR_005502) (Liu et al., 2012). The quality of final contigs was assessed using CheckM (RRID: SCR_016646) v.1.0.5 (Parks et al., 2015) and QUAST (RRID: SCR_001228) v.5.1.0rc1 (Gurevich et al., 2013). The genome sequence was annotated using the NCBI Prokaryotic Genomes Automatic Annotation Pipeline (PGAP) (RRID: SCR_021329) (Tatusova et al., 2016).

To assess the taxonomic status of strain Mr9^T^ as a new taxon, the genome sequence similarities among closely related species were estimated by computing the overall genome relatedness index (OGRI) (Chun and Rainey, 2014). The average nucleotide identity (ANI) was calculated using the OrthoANIu tool (RRID: SCR_022562) (Liu et al., 2016). The digital DNA–DNA hybridization (dDDH) was calculated using the Genome-to-Genome Distance Calculator (GGDC) website, with formula 2 (Meier-Kolthoff et al., 2013). This genomic comparison was between strain Mr9^T^ and type species of validly published species names as well as two genomes of non-type cultivated species of the genus *Leeuwenhoekiella*. The results were evaluated based on the cutoff for species boundaries; ANI, 95–96% (Konstantinidis and Tiedje, 2005; Goris et al., 2007; Richter and Rosselló-Móra, 2009; Chun and Rainey, 2014), and dDDH, 70% (Stackebrandt and Goebel, 1994; Auch et al., 2010).

#### 2.2.3. Phylogenetic and phylogenomic analysis

To infer the 16S rRNA gene-based phylogeny, the respective sequences of strain Mr9^T^ and its close relative species of the genus *Leeuwenhoekiella* were downloaded from DDBJ/ENA/GenBank databases. After sequence alignment, phylogenetic trees were constructed using ARB v.6.0.5 software package (Westram et al., 2011) with neighbor-joining (NJ) (Saitou and Nei, 1987), maximum-parsimony (MP) (Fitch, 1971), and maximum-likelihood (ML) (Felsenstein, 1981) algorithms. The distance matrix was corrected by applying the Jukes–Cantor model of DNA evolution (Jukes and Cantor, 1969). To infer ML phylogeny, the general time-reversible model (Tavaré and Miura, 1986) with gamma distribution and proportion of invariant sites to estimate rate heterogeneity over sites (GTR + Γ + I) were used. Branch support was assessed by 1,000 bootstrap pseudoreplicates (Felsenstein, 1985).

Since phylogenies based on the 16S rRNA gene sequence comparison have been demonstrated not to be totally reliable in determining evolutionary relationships (Corral et al., 2018; de la Haba et al., 2018; Infante-Domínguez et al., 2020), a more robust genome-based phylogeny was inferred by constructing a phylogenomic tree. For this purpose, 11 genome sequences of cultivated species of the genus *Leeuwenhoekiella* were retrieved from the NCBI GenBank database (Supplementary Table 1). Of those genomes, nine corresponded to type strains comprising seven *Leeuwenhoekiella* species (given the fact that two genomes from each of the type strains of *L. palythoae* and *Leeuwenhoekiella marinoflava* were available in the public databases, although with different culture collection designation key); the other two out of 11 genomes corresponded to cultivated non-type strains but closely related to *Leeuwenhoekiella* sp. strain Mr9^T^.

Pan- and core-genome datasets were determined using an all-vs.-all BLAST comparisons among the coding sequences (CDS) and translated CDS features of the annotated genomes under study, as previously described (de la Haba et al., 2019). Then, single-copy core gene and amino acid sequences were individually aligned with muscle (Edgar, 2004) and concatenated into a super-nucleotide or super-protein alignment, which was further analyzed to generate the phylogenomic tree using the appropriate ML algorithm implemented in FastTreeMP v.2.1.8 (Price et al., 2010). Generalized time-reversible (GTR) model of nucleotide evolution (Tavaré and Miura, 1986) and Jones–Taylor–Thornton (JTT) model of amino acid evolution (Jones et al., 1992), both with a single rate for each site (CAT), were applied for nucleotide- and amino acid-based phylogenomic reconstructions, respectively. Tree branch support was inferred using the Shimodaira–Hasegawa test (Shimodaira and Hasegawa, 1999). For the display, annotation, and management of 16S rRNA gene-based and phylogenomic trees, the online tool iTOL (RRID: SCR_018174) v.6.5.2 (Letunic and Bork, 2021) was used.

#### 2.2.4. Phenotypic and physiological characterization

The macroscopic features of *Leeuwenhoekiella* sp. strain Mr9^T^ were based on the colony morphology in solid media SWOM and MA during incubation from the 4th to the 30th day. The colony appearance such as shape, pigmentation, and consistency was visually evaluated. Cell morphology, size, and motility were observed by phase-contrast microscopy (Olympus BX41, Tokio, Japan) from a 72-h liquid culture incubated at 25°C with agitation. The optimal growth conditions and tolerance to temperature, salinity (NaCl), and pH were determined in the following ranges: temperature, 4°C–55°C, intervals of 10°C; NaCl, 0–20% (w/v), intervals of 0.5%; and pH, 3.0–12.0, intervals of 1.0.

The basal medium for salinity and pH tests contained 0.1% (w/v) of yeast extract. For the salinity, the basal medium was prepared in distilled water, and for the pH test, the salinity was adjusted to 3.5% (w/v) of NaCl (sea salts, Sigma-Aldrich, MO, USA) and then adjusted with MES (pH 5.0–6.0), MOPS (pH 6.5–7.0), Tris (pH 7.5–8.5), or CHES (pH 9.0) at a final concentration of 50 mM.

Marine broth (MB) and seawater oligotrophic medium (SWOM) broth were used for temperature testing, both with original formulations to observe the difference in temperature among synthetic and natural media. For each test, 2 mL of the basal medium was inoculated with 100 μL of a 48-h liquid culture (0.5 McFarland scale = 1.5 × 10^8^ CFU/mL). All tubes were incubated with agitation at 25°C, except those for the temperature tolerance test.

The capacity of strain Mr9^T^ to grow under anaerobic conditions was determined by inoculating MA and SWOM agar plates and incubation at 25°C for 72 h in an anaerobic jar with an anaerobic gas generator and anaerobic indicator (Oxoid). Oxidase activity was examined by using oxidase sticks (PanReac AppliChem, Darmstadt, Germany) containing tetramethyl-p-phenylenediamine. Catalase activity was assessed by adding a 3% (w/v) H_2_O_2_ solution to colonies on a solid medium.

The biochemical and physiological tests were evaluated following the methods for characterization tests described by Barrow and Feltham (1993). Hydrolysis of Tween 80, gelatin, and DNA, urease, indole production from tryptophan, methyl red and Voges–Proskauer tests, Simmons’s citrate, production of H_2_S, and anaerobic growth in the presence of nitrate, arginine, or DMSO were evaluated supplementing the medium with 3.5% (w/v) of NaCl. The physiological profiling was approached by testing the assimilation of a battery of carbohydrates, alcohols, organic acids, and amino acids as a sole source of carbon and energy. The basal medium contained 0.05% (w/v) of yeast extract and 3.5% (w/v) of NaCl supplemented with 1% (w/v) of the tested substrate.

For comparative purposes, the type strain of the closest species to *Leeuwenhoekiella* sp. strain Mr9^T^ according to the 16S rRNA gene sequence comparison, *Leeuwenhoekiella nanhaiensis* KCTC 42729^T^, and the type strain of the type species of the genus, *L. marinoflava* Laboratory of Microbiology of Ghent University (LMG) 1345^T^, were purchased from the Korean Collection for Type Cultures (KCTC) and from the Laboratory of Microbiology of Ghent University/Belgian Coordinated Collections of Microorganisms (BCCM), respectively. All phenotypic and physiological tests were determined in triplicate including the reference strains and under the same laboratory conditions. The results were reported after 48–72 h, based on the controls (without inoculum).

#### 2.2.5. Chemotaxonomic analysis

The polar lipid analysis of *Leeuwenhoekiella* sp. strain Mr9^T^ was carried out by DSMZ services, Leibniz Institute DSMZ-German Collection of Microorganisms and Cell Cultures (Germany), from the deposited strain *L. parthenopeia* Mr9^T^ (DSM 112950). In brief, polar lipids were extracted using the modified Bligh and Dyer’s (1959) method and separated by two-dimensional silica gel thin-layer chromatography. Total lipids were revealed by spraying molybdate-phosphoric acid and specific reagents to detect defined functional groups.

The composition of cellular fatty acids methyl esters was also determined following the protocol recommended by the MIDI Microbial Identification System (Sasser, 1990). This determination was carried out at the CECT, Spanish Type Culture Collection (Valencia, Spain). The biomass of strain Mr9^T^ for the fatty acid determination was obtained from a culture on MA after 72 h at 30°C of incubation. The cellular content of fatty acids was analyzed by gas chromatography with an Agilent 6850 gas chromatograph and identified according to the TSBA6 method using the Microbial Identification Sherlock software package (MIDI, 2012).

### 2.3. Relative abundance of *Leeuwenhoekiella* sp. strain Mr9^T^ in marine habitats

To explore the occurrence of the *Leeuwenhoekiella* sp. strain Mr9^T^ in marine microbial communities from different habitats, the genome of this strain was mapped against 41 metagenomes of the Mediterranean Sea. The mapping involved two metagenomic collections from Mediterranean Sea datasets (Supplementary Table 2) derived from the global survey Tara Oceans expedition (Pesant et al., 2015; Sunagawa et al., 2015) and the metagenomic profile study at different depths during seasonal stratification and mixing period along the water column (Haro-Moreno et al., 2017, 2018, 2021; López-Pérez et al., 2017).

The genome sequence of *Leeuwenhoekiella* sp. strain Mr9^T^ was recruited from the metagenomic datasets using BLASTn (RRID: SCR_001598) search, considering only the reads that matched the genome with a nucleotide similarity ≥99% over a minimum alignment length of ≥50 bp, and discarding all recruitments with less than three reads per kilobase of genome per gigabase of metagenome (RPKG) (Haro-Moreno et al., 2018).

Relative abundance was calculated and plotted using the abundance scale from 0.0 to 0.01% for each of the five categories of metagenomic collections: winter; early autumn incubation samples; early autumn depth profile stratification; summer and early autumn; and Tara Oceans Expedition.

### 2.4. Comparative genomic analysis of secondary metabolite biosynthetic gene clusters

The potential of species of the genus *Leeuwenhoekiella* to produce novel compounds was predicted by exploring the secondary metabolite biosynthetic gene clusters (BGCs) encoded in their genomes using antiSMASH (RRID: SCR_022060) v.6.0 (Blin et al., 2021). All genome sequences derived from type strains of *Leeuwenhoekiella*, including *L. parthenopeia* strain Mr9^T^, were analyzed. Cluster sequence similarities were obtained by comparison with experimentally characterized genes encoding the biosynthesis of known chemical molecules present in the Minimum Information about a Biosynthetic Gene cluster (MIBiG) database (Kautsar et al., 2019). BGC relationships among species were circularly displayed using Circos (RRID: SCR_011798) Table Viewer visualization software v.0.63-9 (Krzywinski et al., 2009). In addition to antiSMASH, the antimicrobial potential was evaluated by predicting gene clusters responsible for producing post-translationally modified peptides (RiPPs) and other bacteriocins using the BAGEL 4 web server (van Heel et al., 2018). To determine the BGC homologies with known and patented proteins, each sequence of core biosynthetic gene cluster was translated into amino acids and further searched against non-redundant proteins and patented protein sequences database (pataa) using BLASTP (RRID: SCR_001010). Assignation of proteins to families was carried out using the Pfam (RRID: SCR_004726) database (Mistry et al., 2021).

Biosynthetic gene clusters diversity and evolution across the genus *Leeuwenhoekiella* were assessed by calculating the Jaccard index (JI), adjacency index (AI), and domain sequence similarity (DSS) of each BGC by employing the software BiG-SCAPE (Biosynthetic Gene Similarity Clustering and Prospecting Engine) (RRID: SCR_022561) and CORASON (CORe Analysis of Syntenic Orthologs to prioritize Natural Product Biosynthetic Gene Clusters) (Navarro-Muñoz et al., 2020). Based on sequence similarity networks that encode the biosynthesis of highly similar or identical molecules, BGC sequences from each genome were linked to enzyme phylogenies to create gene cluster families (GCFs). This approach allowed us to obtain a global biosynthetic profile to determine the potential of all species of this genus to produce novel compounds.

### 2.5. Lipidomic bioassay of *Leeuwenhoekiella parthenopeia* Mr9^T^ on tumor cell viability

#### 2.5.1. Total lipid extraction of bacterial cell membrane

As several antitumor compounds have been obtained from marine bacteria, we evaluate the *in vitro* activity of *L. parthenopeia* Mr9^T^ extracts on eukaryotic cells. Since the non-diffusible yellow pigment of strain Mr9^T^ is retained in the cells and low released to the medium, a poor yield of extract not displaying any activity was obtained from the free-cell supernatant. Therefore, the extraction of the total lipidic content of the cell membrane (lipidome) was performed following a modified Bligh and Dyer protocol (Bligh and Dyer, 1959). This protocol allows the extraction of total lipids constituents of cell matrix such as lipopolysaccharides, phospholipids, glycolipids, lipoproteins, carotenoid pigment content, and other medium-to-low polarity compounds included in the cells. As carotenoid pigments are liposoluble, these are extracted together as lipids. In detail, strain Mr9^T^ was cultivated in a mat over SWOM solid medium, and after 7 days, when the pigmentation was more intense, the cell biomass was collected and washed by resuspending it in sterile seawater or 3.5% (w/v) NaCl sterile solution and lately centrifuged.

The pellet was resuspended with 3.5% NaCl solution in equal proportion 1:1 (w/v), that is, to 1 g of pellet, 1 mL of 3.5% NaCl was added to obtain an aqueous cell suspension. A monophasic mixture of cell suspension, methanol, and chloroform was created in a ratio of 0.8:2:1 (v/v). This ratio ensures that all membrane components independent of their affinity (hydrophobic or hydrophilic) are solubilized in a monophase. After gently mixing by inversion for 1 h, the mixture was centrifuged, and the supernatant was separated from the colorless pellet. The extraction from the pellet was repeated and collected from both supernatants. The monophase of supernatants was disrupted by adding 150 μL of KCl 0.2 M. Bilayer phases were obtained after centrifugation, and the lower phase of chloroform was collected in a weighted empty glass vial and dried under a fume hood. A total of 200 μL of chloroform was added to the residual aqueous layer, mixed, and centrifuged again until residual pigments were removed from the suspension. The dried extracts were weighed and stored at −20°C. For screenings, a stock of the extract was prepared by dissolving it in DMSO at a final concentration of 100 mg/mL.

#### 2.5.2. Cell line culture

The lipid extract from *L. parthenopeia* Mr9^T^ was tested against two human tumor cell lines: metastatic prostate adenocarcinoma DU-145 (ATCC HTB-81™) and glioblastoma U-87 MG (ATCC HTB-14™) of moderate and high malignancy, respectively. As a normal cell line, immortalized human keratinocyte HaCaT (RRID: CVCL_0038) was used. All cell lines were cultured in Dulbecco’s Modified Eagle Medium (DMEM) with high glucose, supplemented with inactivated 10% (v/v) of fetal bovine serum (FBS), 100 IU/mL of penicillin G, 100 mg/mL of streptomycin, and 2 mM L-glutamine, and incubated at 37°C in a 5% CO_2_ humidified atmosphere (Zuchegna et al., 2020).

#### 2.5.3. Antiproliferative assay and statistical analysis

The cell viability of tumor and normal cells after treatment with the lipid extract of *L. parthenopeia* Mr9^T^ was assessed by colorimetric MTT [3-(4,5-dimethylthiazolyl)-2,5-diphenyl tetrazolium bromide] assay. In detail, all cell lines were seeded with 3 × 10^3^ cells/well in 96-well microtiter plates and incubated for 24 h at 37°C for cell adhesion. After this time, the medium was replaced with fresh medium with increasing concentration (3.5 to 1,000 μg/mL) of lipid extract of strain Mr9^T^.

For dose–response curve, 9-point serial dilutions (1:2) were performed. The vehicle DMSO without lipid extract (zero drug: 0 μg/mL) was used as a control. Plates were incubated for 24, 48, and 72 h. After each test time, the medium was removed and 100 μL of a 0.5-mg/mL MTT solution was added to each well and incubated at 37°C for 3 h. Then, the MTT solution was gently removed and 100 μL of DMSO was added to dissolve the formazan crystals. The plate was gently mixed for about 2 min under light protection, and the optical density in each well was measured using a microplate spectrophotometer (TECAN Infinite^®^ 200 PRO) at 570 nm. Three individual replicates of the experiment were performed, each time in triplicate.

Statistical analyses were performed using GraphPad Prism Software V9.4.1 (RRID: SCR_002798) v.9.4.1. Data are expressed as a percentage normalized with controls. Values are represented as the mean ± standard deviation (SD) of three independent experiments in triplicate (N 9). Statistical significance between groups was determined by two-way analysis of variance (ANOVA) (mixed model) followed by Dunnett’s multiple comparisons test.

In addition, statistical significance between the different cell lines for each biological replicate (time tested) was determined by two-way ANOVA followed by Tukey’s multiple comparisons test with a single pooled variance [*p*-value in detail: 0.002 (^**^), 0.0002 (^***^), <0.0001 (^****^)]. A *p*-value < 0.05 is considered statistically significant and a *p*-value < 0.0001 is considered statistically highly significant. The IC_50_ value of the total lipid extract (lipidome) was determined by non-linear regression/dose–response inhibition [curve fit (Inhibitor) vs. normalized response].

### 2.6. Dereplication: HPLC/UV/HRMS analysis

Analysis of the composition of the lipidic extract of *L. parthenopeia* Mr9^T^ was performed by liquid chromatography hyphenated to UV detection and high-resolution tandem mass spectrometry (HPLC-UV-HRMS) using a previously reported analytical method (Martín et al., 2014). Nuclear magnetic resonance (^1^H NMR) spectroscopy and heteronuclear single quantum coherence (HSQC) analyses were performed in a mixture of CD_3_OD–CDCl_3_ at 24°C on a Bruker Avance III spectrometer equipped with a 1.7-mm micro-cryoprobe.

## 3. Results

### 3.1. Strain Mr9^T^ is a novel species of the phylum *Bacteroidota*

The use of sampling strategies that consider seasonal and diel factors was the premise of this study to reach the hidden microbiota. From night serial samplings carried out in late autumn 2020, a collection of isolates, including the strain Mr9^T^, was obtained from reef seawater in the gulf of Naples, Italy.

**FIGURE 1:**
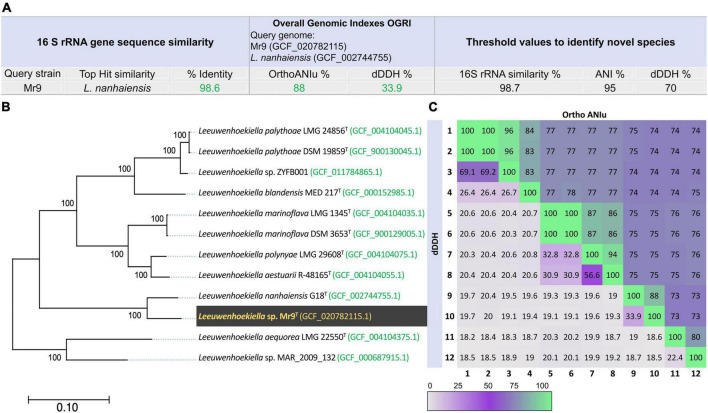
**(A)** 16S rRNA gene sequences similarity and overall genome relatedness index (OGRI) values among strain Mr9^T^ and *Leeuwenhoekiella nanhaiensis* G18^T^. **(B)** Approximately maximum-likelihood phylogenomic tree based on the alignment of 2,103 translated single-copy core genes, showing the relationship among strain *Leeuwenhoekiella* sp. Mr9^T^ and the members of the genus *Leeuwenhoekiella.* The genome sequence accession numbers are shown in parentheses. Bootstrap percentages are shown above the branches. Bar, 0.10 substitutions per nucleotide position. **(C)** Heatmap plot of genome-to-genome analysis by OrthoANIu (upper triangle) and dDDH (lower triangle) showing the percentage of similarity among the genus under study. The colors are displayed according to the color key, with a 100% value when converging in the matrix with the same species.

The physicochemical parameters of seawater in the sampling point registered a temperature of 13.8°C, pH 8.17, TDS of 2,515 ppm, and salinity of 3.4%. The strain Mr9^T^ was first isolated on a natural seawater oligotrophic medium (SWOM) producing smooth and yellow colonies after 4 days of incubation at room temperature (Supplementary Figure 1). According to the 16S rRNA gene sequence comparison, the strain Mr9^T^ was affiliated with the genus *Leeuwenhoekiella*, most closely related to the species *L. nanhaiensis* G18^T^, sharing 98.6% sequence similarity. The genomic similarities inferred by measurements of OGRIs established that strains Mr9^T^ and *L. nanhaiensis* G18^T^ have 88.0% of average nucleotide identity (OrthoANIu) and 33.9% of digital DNA–DNA hybridization (dDDH) (Figure 1A). Species delineation based on a 98.7% for the standard taxonomic marker 16D rRNA gene sequence (Chun et al., 2018), on a 95% ANI (Konstantinidis and Tiedje, 2005; Goris et al., 2007; Richter and Rosselló-Móra, 2009; Chun and Rainey, 2014), and 70% dDDH (Stackebrandt and Goebel, 1994; Auch et al., 2010), strain Mr9^T^ represents a new species of the genus *Leeuwenhoekiella*, since it exhibited percentages to known species below these established thersholds.

The phylogenomic tree (Figure 1B), based on the alignment of 2,103 translated single-copy core genes, clearly showed that *Leeuwenhoekiella* sp. Mr9^T^ forms a separate branch, clustering with *L. nanhaiensis* and it is not related to any other species affiliated with this genus. A similar result was observed in the phylogenetic tree inferred from the alignment of 16S rRNA gene sequences (Supplementary Figure 2). The genome-to-genome relatedness obtained from dDDH and OrthoANIu analysis against the 11 genomes of species of the genus *Leeuwenhoekiella* (Figure 1C) showed that *Leeuwenhoekiella* sp. Mr9^T^ is not related to any described species of this genus, as previously confirmed by the limits for species delineation. Taxonomically, the genus *Leeuwenhoekiella* belongs to the family *Flavobacteriaceae* (Reichenbach, 1992), order *Flavobacteriales*, class *Flavobacteriia* (Bernardet, 2015), and phylum *Bacteroidota* (Oren and Garrity, 2021). Currently, this genus constituted only seven species (Parte et al., 2020), all derived from marine or sea-related habitats.

The differential phenotypic and physiological features of *Leeuwenhoekiella* sp. Mr9^T^ with respect to the closest related species *L. nanhaiensis* and the type species of the genus, *L. marinoflava*, are shown in Table 1. The polar lipid profile obtained by two-dimensional thin-layer chromatography (2D TLC) is shown in Supplementary Figure 3.

**TABLE 1:** Differential features of strains: Mr9^T^, *Leeuwenhoekiella marinoflava* LMG 1345^T^, and *L. nanhaiensis* KCTC 42729^T^.

Feature	Strain Mr9^T^	*L. nanhaiensis* KCTC 42729^T^	*L. marinoflava* LMG 1345^T^
Cell size (width × length; μm)	0.6 × 1.0	0.4–07 × 1.4–4.1	1.0 × 1.5
Colony pigmentation	Yellow/orange	Orange	Yellow
Oxidase	−	−	+
Nitrate reduction	+	−	−
**Hydrolysis of:**			
Aesculin	+	+	−
Casein	−	−	+
DNA	−	+	−
Tween 80	−	+	+
Starch	−	+	−
**Acid production from:**			
L-Arabinose	+	+	−
D-Galactose	+	−	+
Glycerol	−	−	+
D-Glucose	+	+	−
D-Maltose	+	−	−
D-Sucrose	+	−	−
D-Xylose	+	+	−
DNA G + C (mol%, genome)	42.2	42.1	37.5

All data are from this study. +, positive reaction; −, negative reaction.

Based on the results obtained from the 16S rRNA gene sequences similarity, OGRIs, phylogenomic reconstruction, and physiological features, the strain Mr9^T^ constitutes unequivocally a new species of the genus *Leeuwenhoekiella*, for which the name *L. parthenopeia* sp. nov. is proposed. The type strain Mr9^T^ was deposited in three public culture collections: Spanish Type Culture Collection (CECT); Laboratory of Microbiology of Ghent University LMG/Belgian Coordinated Collections of Microorganisms (BCCM); and German Collection of Microorganisms and Cell Cultures GmbH (DSMZ). *L. parthenopeia* Mr9^T^ is available in these culture collections as CECT 30318^T^, DSM 112950^T^, and LMG 32428^T^, respectively.

#### 3.1.1. Description of *Leeuwenhoekiella parthenopeia* sp. nov.

*Leeuwenhoekiella parthenopeia* (par.the.no.pei’a. L. fem. adj. *parthenopeia*, of or belonging to Naples) cells are Gram-stain-negative rods with 0.6 × 1.0 μm; colonies are circular, entire, yellow-to-orange pigmented, with 2–4 mm in diameter in medium MA incubated at 25°C for 7 days; able to grow in oligotrophic medium with 3.0–8.0% (w/v) salts (optimal at 3.5%), in the pH range of 6.5–9.0 (optimal at pH 8.0) and from 10.0 to 37.0°C (optimal at 25°C); chemoorganotrophic and aerobic; and catalase positive and oxidase negative. Nitrate is reduced, but nitrite is not. Indole and H_2_S are not produced. Voges–Proskauer and methyl red are negative. Aesculin is hydrolyzed, but casein, DNA, gelatin, Tween 80, and starch are not hydrolyzed. Acid is produced from L-arabinose, D-galactose, D-glucose, D-maltose, D-sucrose, and D-xylose but not from glycerol. Major fatty acids (>10%) are C_17:0_ iso 3-OH and iso-C_15:0_, followed by iso-C_15:1_ G, iso-C_17:1_ ω9c/C_16:0_ 10-methyl, and C_16:1_ ω6c/C_16:1_ ω7c. The polar lipid profile consists of two aminolipids, one glycolipid, three lipids of undetermined composition, and a phospholipid with similar mobility to phosphatidylethanolamine (PE) as major lipid. The DNA G + C content is 42.2 mol% (genome).

The type strain is Mr9^T^ (=CECT 30318^T^ = DSM 112950^T^ = LMG 32428^T^), isolated from seawater in the Gulf of Naples, Italy. The GenBank/EMBL/DDBJ accession numbers for the 16S rRNA and the complete genome of the type strain are MW785572 and JAJGMW000000000, respectively.

### 3.2. *Leeuwenhoekiella parthenopeia* belongs to the rare biosphere

The occurrence of *L. parthenopeia* Mr9^T^ in marine habitats was mapped against 41 Mediterranean Sea metagenomes categorized into five groups by depths and seasons.

**FIGURE 2:**
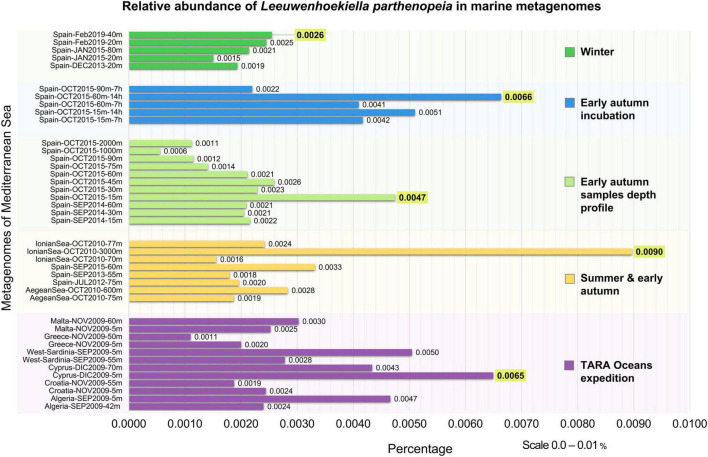
Occurrence of *Leeuwenhoekiella parthenopeia* Mr9^T^ in marine metagenomes from the Mediterranean Sea in the five different categories of metagenomic collections. Y-axis: winter, early autumn incubation, early autumn depth profile stratification, summer and early autumn, and Tara Oceans expedition. X-axis: relative abundance in percentage with the scale from 0.0 to 0.01%. The higher relative abundance for each data block is highlighted in yellow.

As observed in the chart (Figure 2), *L. parthenopeia* showed a relatively higher abundance as follows: 0.0026% at 40-m depth during winter; 0.0066% at 60-m depth after 14 h of incubation of the sample from early autumn, October; 0.0047% at 15-m depth in a non-incubated sample from early autumn depth profile; and 0.0090% at 3,000-m depth across summer and early autumn. Finally, in the TARA Oceans expedition dataset, the higher abundance was 0.0065% at 5-m depth during December.

The results obtained from the metagenomic mapping across depths and seasons showed that *L. parthenopeia* is present in the microbial communities of 41 metagenomes investigated with a maximum detected relative abundance of 0.0090%. The average relative abundance of *L. parthenopeia* Mr9^T^ from all categories of metagenomic data from the Mediterranean Sea was as low as 0.0029%, revealing the scarce occurrence of this bacterium in the total microbial communities analyzed.

### 3.3. Biosynthetic potential of the genus *Leeuwenhoekiella*

#### 3.3.1. Biosynthetic gene cluster analysis of *Leeuwenhoekiella parthenopeia* Mr9^T^

The potential of *L. parthenopeia* Mr9^T^ to synthesize specialized compounds was inferred by predicting the secondary metabolite biosynthetic gene clusters (BGCs) encoded in its genome. Through antiSMASH analysis, the biosynthetic profile of *L. parthenopeia* Mr9^T^ consisted of five BGCs belonging to four typologies of secondary metabolite clusters: two terpenes; one type III polyketide synthase (T3-PKS); one NRPS; and one aryl polyene (APE). As shown in Table 2, just one BGC belonging to the terpene region exhibited a low homology of 28% with a carotenoid biosynthetic gene cluster from *Myxococcus xanthus*, while the rest of the BGC types do not show similarities with any experimentally characterized gene encoding the synthesis of known chemical molecules present in the MIBiG repository (Kautsar et al., 2019). Nevertheless, according to the protein family database Pfam, the amino acid sequence of core enzyme-coding genes of each BGC of *L. parthenopeia* Mr9^T^ was related with multifunctional enzymes as follows: terpene—squalene/phytoene synthase and lycopene cyclase protein; T3-PKS—chalcone and stilbene synthases; NRPS—AMP-binding enzyme/phosphopantetheine attachment site; APE—beta-ketoacyl synthase. Besides the BGCs predicted and their relatedness with protein families, the mining of gene clusters involved in the biosynthesis of bacteriocins, and RiPPs obtained with BAGEL 4 web server revealed two areas of interest in the genome with homologies higher than 97% with gene clusters related with the biosynthesis of Zoocin A (bacteriocin) and Sactipeptides (RiPP), both involved in the synthesis of natural complex products.

**TABLE 2:** Biosynthetic gene cluster types of *Leeuwenhoekiella parthenopeia* Mr9^T^ showing its relatedness with known molecules, protein families hits, and patented proteins based on sequence similarity.

BGC’s content			
**Prediction tool: AntiSMASH**			
**Type**	**Molecule related similarity (MIBiG)**	**Protein family identity (Pfam hit)**	**Patented protein sequences (pataa) similarity**
Terpene	Carotenoid 28%	PF00494.19 Squalene/phytoene synthase	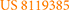 48.02% (Polynucleotides and polypeptides for pharmaceutical, agricultural, cosmetic, and nutraceutical context)
	Unknow	PF05834.12 Lycopene cyclase protein	US 7630836 34.69% (Polynucleotide array)
Aryl polyene	Unknow	PF00109.26 and PF02801.22 Beta-ketoacyl synthase, N and C terminal domain	US 6673910 34.95% (Aminoacidic sequences for diagnostics and therapeutics of *Moraxella catarrhalis*)
T3-PKS	Unknow	PF00195.19 and PF02797.15 Chalcone and stilbene synthases, N and C terminal domain	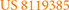 67.08%
NRPS-like	Unknow	PF00501.28 AMP-binding enzyme PF00550.25 Phosphopantetheine attachment site	US 7319142 22.39% (Insecticidal proteins for controlling insect infestation)
**Bacteriocins and RiPPs content**			
**Prediction tool: BAGEL4**			
**Class Gene name**	**Function**	**Protein homology redundant**	**Patented protein sequences (pataa) similarity**
(RiPP) Sactipeptides BmbF	GTP 3′,8-cyclase Peptoclostridium acidaminophilum	GTP 3′,8-cyclase MoaA 97.21%	US 11078247 43.34% (Insecticidal proteins for controlling insect infestation)
(Bacteriocin) Zoocin A	Zoocin A	Peptidase M23 97.36%	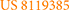 50.66%

The best score for amino acidic homology with patented protein sequences is marked in orange.

Furthermore, amino acid sequence homology with patented protein sequences (pataa) showed that the biosynthetic gene content of *L. parthenopeia* Mr9^T^ does not exhibit full similarity to any registered patent for various applications, showing a frequent match with patent US 8119385 that falls into the categories of terpene (carotenoids), T3-PKS, and bacteriocin. The identity percentages with five different patents ranged from 22.39 to 67.08%, thus revealing the high potential of *L. parthenopeia* Mr9^T^ for the synthesis of novel polypeptides for biotechnological application.

#### 3.3.2. Diversity and distribution of BGCs across *Leeuwenhoekiella*

In the same way as the profile of *L. parthenopeia* Mr9^T^, the specialized metabolite potential of all species of the genus *Leeuwenhoekiella*, including *L. parthenopeia* Mr9^T^, was assessed by predicting the BGC content and diversity in each of the eight species through antiSMASH. A content of three to four BGCs was identified in each species, except *L. parthenopeia* Mr9^T^, which harbors five BGCs as previously detailed (Supplementary Figure 4). A total of 30 BGCs (29 + 1 hybrid) were identified across the genus and, like the BGC profile of *L. parthenopeia* Mr9^T^, only one of the two BGCs terpenes typologies exhibited 28% homology to a carotenoid gene cluster of *M. xanthus* (Supplementary Table 3). Moreover, this type of terpene is the unique gene cluster present in all species that shows homology with known BGCs; however, its similarity is very low and probably would not code for the synthesis of the same carotenoid as that of *M. xanthus.* The rest of the BGCs types showed no similarities to any experimentally characterized gene encoding the synthesis of known chemical molecules present in the MIBiG repository.

**FIGURE 3:**
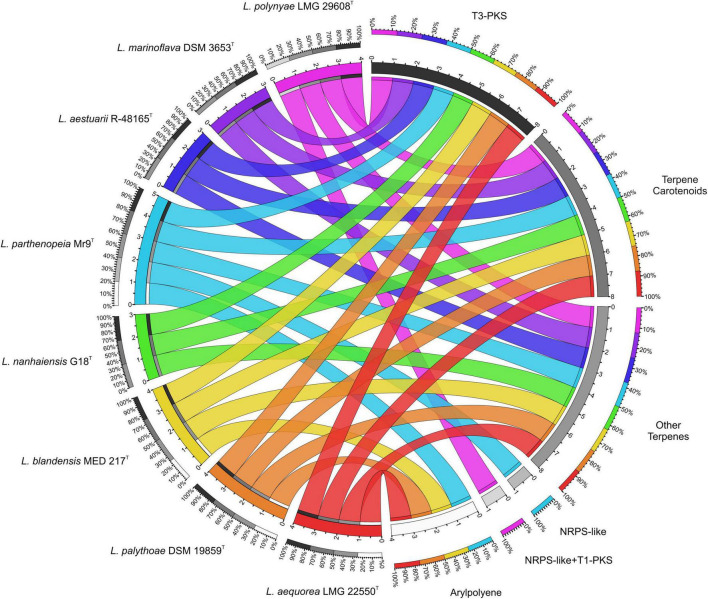
Circle plot showing the relationships among the eight species of *Leeuwenhoekiella* (left half of the circle) and the biosynthetic gene cluster (BGC)’s diversity by typologies (right half of the circle). Each genome is represented by different colors, which are segmented according to the number of BGC content in their genomes. The chords connect both sides indicating, according to their width, the number of BGCs belonging to each typology. The outer rings in the gray scale represent the percentage of presence of BGC and in the color scale the proportion of genomes per BGC.

To determine how the gene cluster profile varies across *Leeuwenhoekiella*, a comparative genomic study was performed to show the correlation between species and categories of BGC. Through the chord diagram of Figure 3, it can be observed that three types of gene clusters are conserved in all eight species of the genus: two terpenes and one T3-PKS. It can also be evidenced by the different BGC contents between *L. parthenopeia* Mr9^T^ and the most closely related species *L. nanhaiensis* G18^T^, where in the latter, the gene clusters NRPS and APE are absent. The exclusive characteristic of *L. parthenopeia* Mr9^T^ BGC pattern with respect to the other species of the genus is the presence of the NRPS gene cluster, which is absent in the rest of the species. Although an NRPS is also present in *L. polynyae*, it constitutes a hybrid NRPS-like + T1-PKS cluster, since both BGCs overlap in the same region of the genome, therefore counting separately within its category.

#### 3.3.3. BGC similarity networking and evolution of the genus *Leeuwenhoekiella*

To evaluate the diversity and evolutionary history of BGC repertoires across members of the genus *Leeuwenhoekiella*, a comparative interspecies analysis of conserved-coding genes was performed by means of sequence similarity networking and BGC phylogenetic reconstruction *via* BiG-SCAPE/CORASON (Navarro-Muñoz et al., 2020).

**FIGURE 4:**
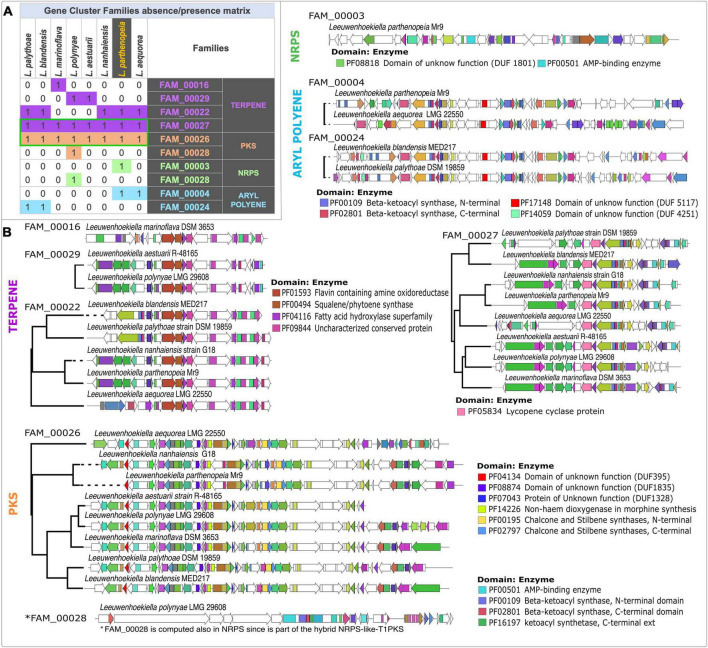
**(A)** Similarity network matrix based on the absence and presence of gene cluster families from each species of the genus *Leeuwenhoekiella*. Green boxes, gene cluster families (GCFs) are present in all members of *Leeuwenhoekiella*. **(B)** Multi-locus approximately maximum-likelihood trees based on the conserved core common domain.

The similarity networking groups BGCs of each species into gene cluster families (GCF) according to the presence, copy number, organization, and sequence homology of the protein domains on antiSMASH-predicted regions. As shown in the similarity matrix calculation obtained through BiG-SCAPE (Figure 4A), the clustering of the total 30 BGC content of *Leeuwenhoekiella* gave rise to nine GCFs affiliated with four types of BGCs, the terpene category being the most represented. It is observed in the matrix that all eight species share the same core enzyme-coding genes that form two families: FAM_00027 and FAM_00026. These GCFs are affiliated with the BGC types terpene and T3-PKS, respectively. Conversely, three singletons were identified: FAM_00016 belonging to terpenes corresponding to *L. marinoflava*; FAM_00003 belonging to NRPS which was exclusive of *L. parthenopeia*, and FAM_00028 presents in *L. polynyae;* the latter corresponds to the chemical hybrid NRPS-like-T1-PKS, which explains the two times computing and its assignation to two categories, NRPS and PKS. These singletons result when the domains present in the BGC do not reach the thresholds that capture sequence similarity, synteny, or presence–absence of the Jaccard index against BGCs from other species to form a network, thus, a family of shared gene groups could not be defined, remaining as an individual member. In all cases, whether as clusters or singletons, after the GCF’s comparison with MIBiG reference BGCs, none of the nine GCFs match any pathways responsible to produce known compounds.

Evolutionary relationships between BGCs and within GCF were initially inferred by CORASON inside the GCF defined for BiG-SCAPE for each family. CORASON establishes phylogenies based on BGC’s conserved core of common domains. The clades resulting from the phylogenetic reconstruction may be responsible for the biosynthesis of different types of chemistry due to the association of specific types of genes encoding additional enzymes. In correspondence with the families in the presence–absence matrix, the phylogenetic reconstructions of all BGCs from *Leeuwenhoekiella* (Figure 4B) generated six multi-locus approximately maximum-likelihood trees, and the three singletons (FAM_00016, FAM_00003, and FAM_00028) do not form clades. The featured conserved core domains and their related enzymes used to define the GCFs are specified for each tree. In addition, the genes that encode functionally uncharacterized proteins (domains of unknown function, DUF) were identified to see their cryptic potential of novel structures and functions.

To further investigate possible distant phylogenetic relationships among these families, CORASON was run outside the BiG-SCAPE-predicted BGC families. Instead of restricting the analysis to anti-SMASH-predicted regions, full *Leeuwenhoekiella* genomes were used as CORASON inputs. As a result, families FAM_00016 (*L. parthenopeia*), FAM_00022 (*L. nanhaiensis, L. marinoflava, L. blandensis, L. palythoae, and L. aequorea*), and FAM_00029 (*L. polynyae and L. aestuarii*) were clustered in a single phylogenetic tree (Supplementary Figure 5).

With this observation, it is shown that a new superfamily that comprises a terpene BGC in each of the species is studied here. It is possible that due to the variation in gene content and sequence similarity, BiG-SCAPE split this superfamily into the three families shown in the presence–absence matrix, but these families shared a common evolutionary history as can be seen in Figure 4B. The same is true for aryl polyene families FAM_00004 and FAM_00024, and they share a common ancestor. In fact, though not all the genomes have an antiSMASH region predicted, the beta-ketoacyl synthase genomic vicinity is conserved in all eight genomes as can be seen in the Supplementary Figure 5 produced by CORASON.

According to the featured conserved core domains linked to enzymes, within the terpene category, the GCF: FAM_00016 (singleton), FAM_00029, and FAM_00022 encode the biosynthesis of highly similar or identical molecules to flavin, squalene, chalcones–stilbenes, and an unknown compound associated with an uncharacterized conserved protein. This category highlights the FAM_00027 which is composed of all species of *Leeuwenhoekiella* revealing the potential of members of this genus to synthesize carotenoid pigments as lycopene.

In the category PKS, the FAM_00026 is also clustered with all species of *Leeuwenhoekiella* and encodes the biosynthesis of carotenoids, chalcone, and stilbenes. The singleton FAM_00028 (*L. polynyae*) also falls in this group, which is duplicated in the category NRPS, since *L. polynyae* contains the hybrid gene cluster NRPS-like-T1_PKS, computing in both groups. The featured conserved domains of this singleton are linked to the family protein AMP-binding enzyme involved in the synthesis of several bioactive compounds and beta-ketoacyl synthase involved in the synthesis of fatty acids. In both cases, given the high capacity to synthesize a wide spectrum of molecules, it is not possible to precise the most probable-related molecule. Regarding the NRPS category, the singleton FAM_00003 (*L. parthenopeia* Mr9^T^) possesses the domain of the unknown function (DU1801) and a major facilitator superfamily domain; in both cases, the possible synthesis of a determined molecule is unknown.

Finally, in the category of aryl polyene (APE), two GCFs, FAM_00004 and FAM_00024, possess common conserved domains of unknown functions (DUF 5117 and DUF 4251) for which the eventually synthesized molecules remain unknown, and the beta-ketoacyl synthase is involved in the synthesis of fatty acids. The GCF analysis was performed, allowing us to detect the frequent presence of domains of unknown function (DUFs) in all GCFs by categories, strongly suggesting that this genus harbors the potential to synthesize novel compounds not described nor chemically tested yet.

### 3.4. Total lipid extract from *Leeuwenhoekiella parthenopeia* affects viability of tumor cells

The potential antiproliferative effect of total lipid extract (TLE) from *L. parthenopeia* Mr9 was evaluated using the colorimetric MTT assay, which measures cellular metabolic activity as an indicator of cell viability. Two human tumor cell lines: metastatic prostate adenocarcinoma DU-145 and glioblastoma U-87 MG of moderate and high malignancy, respectively, were tested. As a normal cell line, it was used to immortalize human keratinocyte HaCaT.

**FIGURE 5:**
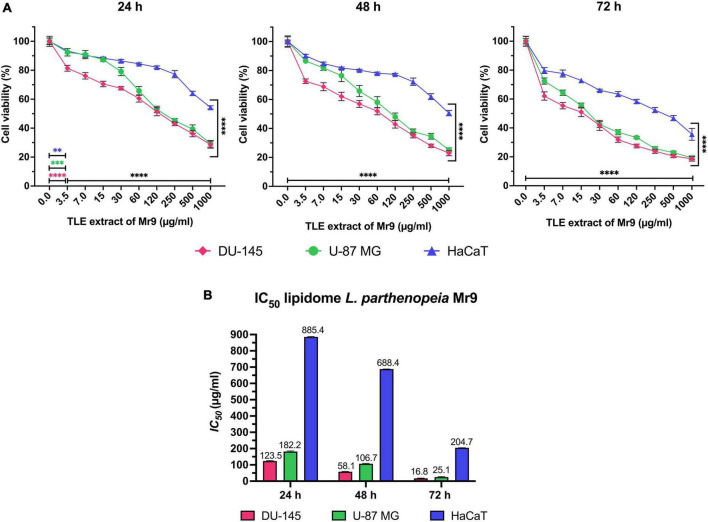
**(A)** Effect of total lipid extract from *Leeuwenhoekiella parthenopeia* Mr9^T^ cell extract in the cell viability of DU-145, U-87 MG, and HaCaT after 24, 48, and 72 h of stimulation with gradual doses. Untreated cells (0 μg/mL) were used as control and indicate cells treated with dimethyl sulfoxide (DMSO) without *L. parthenopeia* Mr9^T^ extract. Control corresponds to 100% of viability. The histograms represent the mean ± SD of three independent experiments performed in triplicate (*n* = 9), normalized to untreated control cells. Statistical analysis: *p*-value: 0.002 (^**^), 0.0002 (^***^), and <0.0001 (^****^) compared to the control. **(B)** *IC_50_* inhibitory concentrations of the total lipid extract (TLE) required to inhibit DU-145, U-87 MG, and HaCaT after 24, 48, and 72 h of stimulation.

After the treatment of the cell lines with several doses of TLE from 3.5 to 1,000 μg/mL for 24, 48, and 72 h, it was possible to observe that the viability of tumor cells DU-145 and U-87 MG decreases gradually in time- and dose-dependent manner with lower effect on HaCaT (Figure 5A). In particular, the cell death of DU-145 and U-87 MG above 50% occurred with 120 μg/mL of extract after 24 h, while the cell death of HaCaT happens only with the highest concentration (1,000 μg/mL). After 48 h, DU-145 and U-87 MG are inhibited above 50% with 60 μg/mL, while HaCaT requires the highest concentration of the extract. In all cell lines, both tumor and normal, the greatest inhibitory effect was observed after 72 h of treatment, where the cell viability of DU-145 and U-87 MG decreased significantly, requiring a concentration of around 15 μg/mL to inhibit the 50% of the cells, while HaCaT requires more than 250 μg/mL. Between the two tumor cell lines, DU-145 and U-87 MG, it is observed that TLE is less effective on U-87 MG at doses lower than 60 μg/mL at 24 and 48 h of treatment, and successively at higher concentration, the difference of the inhibitory effect on both tumor cell line is reduced.

According to the cell viability results and the inhibitory concentration value IC_50_ (Figure 5B), the lipid extract of *L. parthenopeia* Mr9^T^ possesses inhibitory activity against metastatic prostate adenocarcinoma DU-145 and glioblastoma U-87 MG with greater effectiveness on DU-145. The minor effect on immortalized human keratinocyte HaCaT suggests a selective effect on tumor cells.

### 3.5. Dereplication: Total lipid composition

**FIGURE 6:**
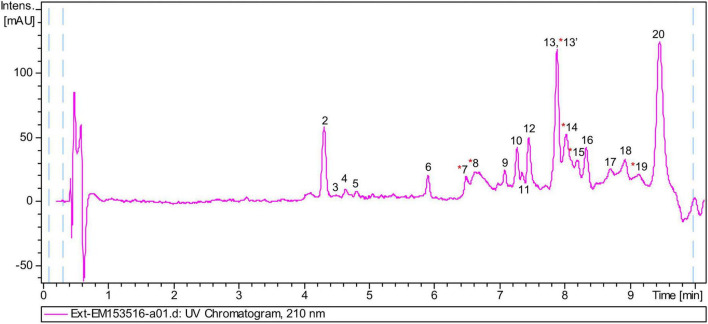
Liquid chromatography and high-resolution tandem mass spectrometry analysis of the lipidic extract from *Leeuwenhoekiella parthenopeia* Mr9^T^ showing 20 peaks in the chromatogram. Red asterisk, known components.

The chemical composition of the lipid extract from *L. parthenopeia* Mr9^T^ was analyzed by HPLC-UV-HRMS, and the molecular formulae of major components were determined. Figure 6 shows the LC/UV trace obtained in the analysis. Searches of molecular formulae of the peaks in the figure against the Dictionary of Natural Products Database (DNP) tentatively identified some compounds and other molecules present in the extract with no matches in the database.

Following the above spectra, 20 peaks were identified, and their molecule formula and putative component are detailed in Table 3. From these peaks, nine molecular formulae do not match in the DNP, five corresponds to fermentation medium component (FMC), and six corresponds to known molecules as follows: sulfobacin-like component, sulfobacin A, WB 3559A, WB 3559B, docosenamide, and topostin B-567.

**TABLE 3:** Peaks with molecule formula and putative components.

Peak	Molecular formula	Putative dereplicated component
1, 2	C_22_H_26_O_6_	Fermentation medium component
3	C_15_H_31_NO_2_	No coincidences in the DNP
4	C_21_H_33_N_3_O_3_	Fermentation medium component
5	C_14_H_18_O_5_	Fermentation medium component
6	C_21_H_37_N	Fermentation medium component
7	C_33_H_67_NO_6_S	Sulfobacin like component
8	C_34_H_69_NO_6_S	Sulfobacin A
9	C_33_H_50_O_8_P_2_	Fermentation medium component
10	C_35_H_68_O_6_S	No coincidences in the DNP
11	C_36_H_70_N_2_O_6_	No coincidences in the DNP
12	C_37_H_72_N_2_O_6_	No coincidences in the DNP
13	C_33_H_50_O_7_P_2_	No coincidences in the DNP
13′	C_36_H_66_N_2_O_7_	WB 3559A
14	C_37_H_68_N_2_O_7_	WB 3559B
15	C_22_H_43_NO	Docosenamide
16	C_33_H_60_O_3_S_2_	No coincidences in the DNP
17	C_35_H_62_O_3_	No coincidences in the DNP
18	C_53_H_58_O_7_P_2_	No coincidences in the DNP
19	C_34_H_65_NO_5_	Topostin B-567
20	C_33_H_52_O_6_P_2_	No coincidences in the DNP

**FIGURE 7:**
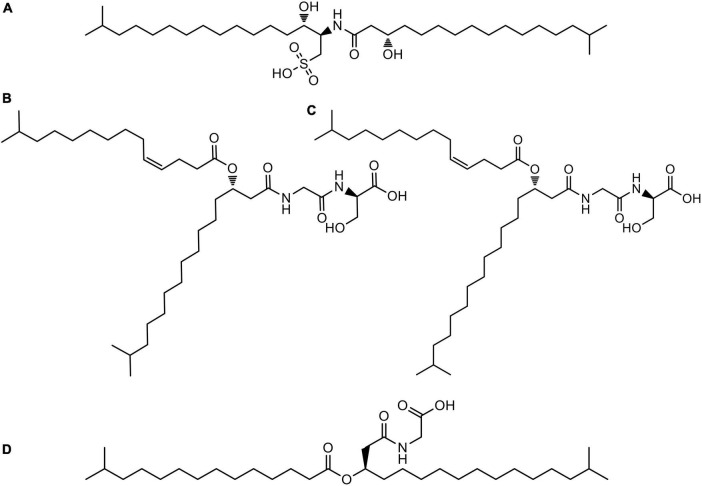
Structures of compounds tentatively identified in the liquid chromatography hyphenated to UV detection and high-resolution tandem mass spectrometry (HPLC-UV-HRMS) analysis of the lipidic extract of *Leeuwenhoekiella parthenopeia* Mr9^T^: **(A)** sulfobacin A; **(B)** WB 3559A; **(C)** WB 3559B; **(D)** topostin B-567.

Searches of molecular formula of the peaks in the figure against the Dictionary of Natural Products Database (DNP) tentatively identified some compounds and other molecules present in the extract with no matches in the database. Figure 7 represents the structures of the compounds tentatively identified in the lipidic extract. Not surprisingly, these compounds present in their structure units of C_17:0_ iso 3-OH, C_15:0_ iso, and C_15:1_ iso fatty acids are identified as major fatty acids in Section “Description of *Leeuwenhoekiella parthenopeia* sp. nov.” NMR analysis corroborated the presence of iso-fatty acids as the major components in the lipid extract.

## 4. Discussion

### 4.1. Isolation and ecology

The isolation of *L. parthenopeia* strain Mr9^T^ from a night seawater sample in autumn supports previous studies on the dynamic and seasonal effect on the microbial population. This isolation could be associated with the natural phenomenon of the DVM and the mixing of the water itself during the cold months since these favor the ascent toward the surface of planktonic particles and solid matter, acting as vectors of the hidden microbiota. The particulate was ascertained by the visual observation and the measurement of TDS that showed high detection in night samples. Genomic and phenotypic features of this strain showed that it constitutes a new species of the genus *Leeuwenhoekiella*, for which we propose the new name *L. parthenopeia* sp. nov.

It is probable that *L. parthenopeia*, as a member of the *Flavobacteriia*, is trapped in small particles such as marine picoplankton, where this class is dominant and typically found at depth (Kirchman et al., 2003; Alonso et al., 2007; Gómez-Pereira et al., 2010; Mestre et al., 2017). In cold waters, a high abundance of some members of *Flavobacteriia* has also been reported (Ghiglione and Murray, 2012; Grzymski et al., 2012), and, in fact, two species of *Leeuwenhoekiella*, *L. aequorea* (Nedashkovskaya et al., 2005) and *L. polynyae* (Si et al., 2015), were isolated from Antarctic Sea water, which also explains that cold seasons would favor the presence of microbial population minorities as a rare biosphere. The lack of isolates of microorganisms belonging to this genus or any member of the phylum *Bacteroidota* in day samples, besides the reduction of particles, demonstrates that the cultivation of *L. parthenopeia* as well as other members within this phylum is not causality and indicates a close association with planktonic particulate, organic matter, algae or neustons colonizing their surface acting as ectosymbiont (Kirchman, 2002; Fernández-Gómez et al., 2013; Mestre et al., 2017; Baumas et al., 2021). Thus, the probability of isolation of similar bacteria may depend on the composition of phytoplankton and temporal dynamics (Rooney-Varga et al., 2005; Teeling et al., 2016; Camarena-Gómez et al., 2021).

Physiological characteristics showed that *L. parthenopeia* has a heterotrophic and versatile metabolism, assimilating various substrates as a carbon and energy source, and suggesting that this bacterium can thrive in a wide range of nutritional circumstances in its habitat. This versatility highly contributed to its fast laboratory domestication, exhibiting the ability to grow in synthetic marine media without requiring natural seawater in culture media as the main component of primary cultures. Its role in nature might fulfill its function as a heterotroph, that is, degrading organic matter (Alonso et al., 2007; Gómez-Pereira et al., 2010; Xue et al., 2020).

The almost undetectable genomic recruitment patterns of the new species *L. parthenopeia* when mapping the 41 metagenomes from the Mediterranean Sea revealed that this species is poorly represented with a mean relative abundance as low as 0.0029%, considering that members of the class *Flavobacteriia* (phylum *Bacteroidota*) are prominent in marine environments across different seasons and depth, accounting from 4.7 to 13.9% (Cram et al., 2015) and from 3 to 20% in planktonic particles (Mestre et al., 2017; Baumas et al., 2021). This infimum percentage reveals the scarce occurrence of this novel bacterium in the total microbial communities analyzed. Based on the arbitrary threshold of 0.1 or 0.01% of relative abundance for defining rare biosphere (Pedrós-Alió, 2012), *L. parthenopeia*, similar to *L. blandensis* (Pedrós-Alió, 2013), is part of the low-abundant fraction of microbial population and therefore belongs to the rare biosphere.

According to all the categories of metagenomes analyzed, we could not observe a pattern of distribution that allows us to establish the depth or the specific season at which *L. parthenopeia* is relatively more abundant. However, we can affirm that despite its scarcity, *L. parthenopeia* is widely distributed in the Mediterranean Sea. A remarkable fact is the highest abundance of *L. parthenopeia* found in the Ionian Sea metagenome, at a depth of 3,000 m, which might suggest that *L. parthenopeia* could be more abundant at abyssal depths. In fact, its closest relative, the species *L. nanhaiensis*, was isolated at a depth of 2,000 m (Liu et al., 2016), while a culture-independent study about the depth and seasonal distribution of *Bacteroidota*, showed that the members of the genus *Leeuwenhoekiella* were detected in deep marine waters (Díez-Vives et al., 2019), therefore, supporting that vertical migration besides the mixing waters are determinant factors to reach the rare microbiota. Our study shows that the sample pre-incubation increases the abundance of *L. parthenopeia*, as observed in the metagenomes from incubated samples collected in October, where the presence of *L. parthenopeia* is doubled, especially after 14 h with respect to the other metagenomes studied. This result indicates that the pre-incubation of the sample before the culturing increases the possibility of isolation of poorly represented species. However, this procedure would contribute equally to the overgrowth of representatives of the most abundant phyla of the marine microbial community, such as *Pseudomonadota* (formerly *Proteobacteria*) and *Cyanobacteria* (Fernández-Gómez et al., 2013).

### 4.2. Biosynthetic potential

The biosynthetic gene profiling of *L. parthenopeia* Mr9^T^ and all species of the genus *Leeuwenhoekiella* showed that members of this genus have in common three BGCs: two terpenes and one T3-PKS. Of these, only one BGC terpene showed a low homology of 28% with a carotenoid molecule, while the rest of the BGCs do not exhibit similarity with any experimentally characterized gene encoding the synthesis of known chemical metabolites. Even though most of the gene clusters did not show a match with known molecules, the nucleotide translated sequences of those BGCs revealed that this genus, including *L. parthenopeia* Mr9^T^, possesses multifunctional enzymes related to the synthesis of squalene, lycopene, fatty acids, flavonoids, and resveratrol; thus, they are probably synthesizing these types of molecules. In contrast to the rest of the species, only *L. parthenopeia* Mr9^T^ possesses NRPS and the significance of this type of BGC relies upon its well-known synthesis of antibiotic and antitumor compounds, thus the importance of *L. parthenopeia* Mr9^T^ with respect to the other species.

Additionally, the low homology of the core peptide with patented proteins confirms that *L. parthenopeia* Mr9^T^ does not synthesize the same proteins that those patented, suggesting its high metabolic capability to produce novel compounds with entirely different biotechnological applications.

Overall, the biosynthetic profile of species of *Leeuwenhoekiella* is very streamlined in comparison with the profile of the species of *Salinispora*, the abyssal marine bacteria producer of antitumor compounds, that harbor 176 distinct BGCs, of which only 24 have been linked to their products (Letzel et al., 2017). Despite this simplicity and given the novelty of these BGCs, they are probably synthesizing molecules with potentially different functionalities.

The similarity networking and evolutive clustering of the BGCs linked to enzyme phylogenies revealed that all species of *Leeuwenhoekiella* form four prominent gene cluster families (GCF) groups and according to this classification, species of this genus synthesize molecules such as flavine, lycopene, and squalene/phytoene. Moreover, the presence of uncharacterized conserved proteins and beta-ketoacyl synthase also suggests the synthesis of molecules of unknown function by species of this genus. Beyond these GCFs, also in evolutionary terms, *L. parthenopeia* Mr9^T^ remains again potentially more interesting than its peers, since the presence of NRPS, which feature domains AMP-binding enzyme, not shared with the other species, confirms that this bacterium may synthesize other compounds of biotechnological interest.

### 4.3. Biological activity: Lipidomic bioassay

Regarding the antiproliferative effect that exhibits the total lipid extract (TLE) from *L. parthenopeia* Mr9^T^ against prostate adenocarcinoma cell line, DU-145, and glioblastoma cell line, U-87 MG, it is important to take into account the nature of these cell lines, considered as moderate and high malignancy, respectively. In fact, the total lipid extract of *L. parthenopeia* proved to be more effective on prostate cells DU-145 than on glioblastoma cells U-87 MG. This result could be expected due to the intrinsic characteristic of the U-87 MG cell line, whose heterogeneous nature is associated with clonal plasticity that makes glioblastoma extremely resistant to current treatments (Osuka and Van Meir, 2017; Oliver et al., 2020). The antecedent is that glioblastoma is one of the most aggressive types of brain cancer, characterized by its rapid multiplication and invasion (Oraiopoulou et al., 2017), which could explain the low effect of the lipid at doses lower than 60 μg/mL at 24 and 48 h, as shown in the line graph of MTT results (Figure 5A). The primary cell line U-87 MG (ATCC HTB-14) has a double time of generation ranging from 24 to 29 h (Kato et al., 2011) and this faster multiplication was also observed under laboratory conditions, for which higher doses of extract and a long exposure time are required. The lipid extract was more effective on DU-145 causing a homogeneous decrease of cell viability from the first 24 h of treatment. In both tumor cell lines, DU-145 and U-87 MG, the cell viability gradually decreased in a time- and dose-dependent manner reaching a maximum efficiency at 72 h. The lower effect of the extract in immortalized human keratinocyte HaCaT, used as no tumor cell line, suggests a selective effect on tumor cells. Individual screenings with purified fractions will allow us to determine if the cell death is caused by a single compound or if it is a synergistic effect of individual compounds, then it will be necessary to identify the molecular mechanism that induces death. In any case, the lipid extract of the cell membrane of *L. parthenopeia* Mr9^T^ possesses an effective antiproliferative activity on tumor cell lines DU-145 and U-87 MG, not previously reported in rare bacteria.

### 4.4. Lipidome analysis

The chemical composition of the lipid extract from *L. parthenopeia* Mr9^T^ consisted of four putative compounds with known chemical structures, besides nine molecules without coincidences in the dictionary of natural products (DNP) identified after the dereplication. Among the known molecules are: sulfobacin A (Kamiyama et al., 1995), WB 3559A, WB-3559B (Yoshida et al., 1985), and topostin B-567 (Suzuki et al., 1990), all are derived from species of the class *Flavobacteriia*, except topostin B-567 isolated from a culture broth of *Flexibacter topostinus* belonging to the class *Cytophagia*. Regarding their biological activities, sulfobacin A is a sulfonolipid that was described as an antagonist of von Willebrand factor receptor (vWF), a blood glycoprotein involved in hemostasis and thus proposed as an antithrombosis agent (Kamiyama et al., 1995). Furthermore, sulfonolipid analogous (flavocristamide A and B) were found to have inhibitory activity against DNA polymerase α in eukaryotic cells (Kohayashi et al., 1995). Subsequently, the cytotoxic properties of this molecule were reported against four cancer cell lines with maximum activity against human mammary adenocarcinoma (Chaudhari et al., 2009). Concerning the compounds WB-3559 A and B, they were reported as potent fibrinolytic agents produced by *Flavobacterium* sp. No. 3559, which stimulates the euglobulin clot lysis time of rabbit plasma (Uchida et al., 1985; Yoshida et al., 1985). Docosenamide, also known as erucamide is a fatty acid amide belonging to a family of brain lipids that induce sleep (Cravatt et al., 1995). This molecule acts as a human metabolite, and it is more used in the neuroscience area. Finally, Topostin B-567 is an inhibitor of mammalian topoisomerase I and was isolated from a broth culture of *F. topostinus* B-572 (Ikegami et al., 1990; Suzuki et al., 1990; Brinkmann et al., 2022). Topoisomerase I is the molecular target for anticancer drugs since the activity of this enzyme is increased in tumor cells (Buzun et al., 2020). Topoisomerase suppressors constitute a family of antitumor agents with cytostatic effect, which mechanism of action is the inhibition of enzyme activity leading to the interruption of DNA strands and causing cell death (Bailly, 2000; Galluzzi et al., 2015). Considering the biological activities of the known compounds, we can hypothesize that the antiproliferative effect of DU-145 and U-87 MG observed *in vitro* in this study could be attributed to sulfobacin A and topostin B-567. The reduction of cell viability could be due to the action of both compounds since both inhibit DNA replication. However, the only one that does so selectively with tumor cells is topostin B-567, which being a specific suppressor of the enzyme topoisomerase I would explain the death of tumor cells and the low effect on epithelial tissue cells. Beyond the interesting known molecules found in the lipid extract, the great potential of *L. parthenopeia* Mr9^T^ remains in the nine molecules without coincidences in the dictionary of natural products and still to be chemically elucidated.

Identifying the active fractions and their chemical structure will allow us to decipher the possible genes involved in the antiproliferative activity shown *in vitro.* Thus, based only on the *in silico* study, we cannot accurately demonstrate which of the BGCs present in the genome of *L. parthenopeia* Mr9^T^ can be responsible for this activity. Nonetheless, the simplicity of the biosynthetic profile of *L. parthenopeia* would make feasible the discrimination of the genes involved in the antitumor activity circumscribed to the four categories of BGC of its profile.

In conclusion, our study demonstrates that the new bacterium *L. parthenopeia* and the other species of the genus *Leeuwenhoekiella* contain biosynthetic genes with potential antiproliferative activities and encourage to carry out similar approaches to explore the marine rare biosphere as a promising source of bioactive compounds.

## Data availability statement

The datasets presented in this study can be found in online repositories. The names of the repository/repositories and accession number(s) can be found in the article/Supplementary material.

## Author contributions

GG, RH, JM, FR, and PC: formal analysis. GG and PC: investigation. GG, RH, JM, FR, CS-P, AF, CZ, SG-F, NS-M, and PC: methodology. GG, RH, JM, SG-F, NS-M, and PC: software and visualization. GG: data curation and writing original draft preparation. RH, FR, CS-P, MV, ER, NS-M, AV, and PC: validation. RH, AV, and PC: writing – review and editing. CS-P, AV, and PC: funding acquisition, project administration, and resources. PC: conceptualization and supervision. All authors contributed to the manuscript revision, read, and approved the submitted version.
