# Supplementary material for: Genomic study and lipidomic bioassay of Leeuwenhoekiella parthenopeia: A novel rare biosphere marine bacterium that inhibits tumor cell viability
Source: Front Microbiol. 2023 Jan 6;13:1090197. doi: 10.3389/fmicb.2022.1090197 (PMC9859067; doi:10.3389/fmicb.2022.1090197)
Supplement: Supplementary file 1 [file Data_Sheet_1.PDF]

Supplementary Material

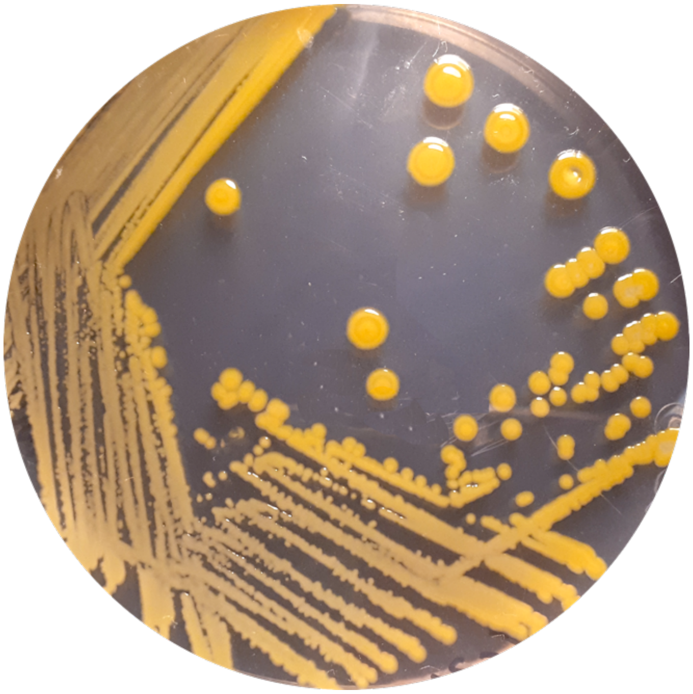

Supplementary Figure 1. Colonies of *Leeuwenhoekiella parthenopeia* Mr9 in Zobell marine agar 2266.

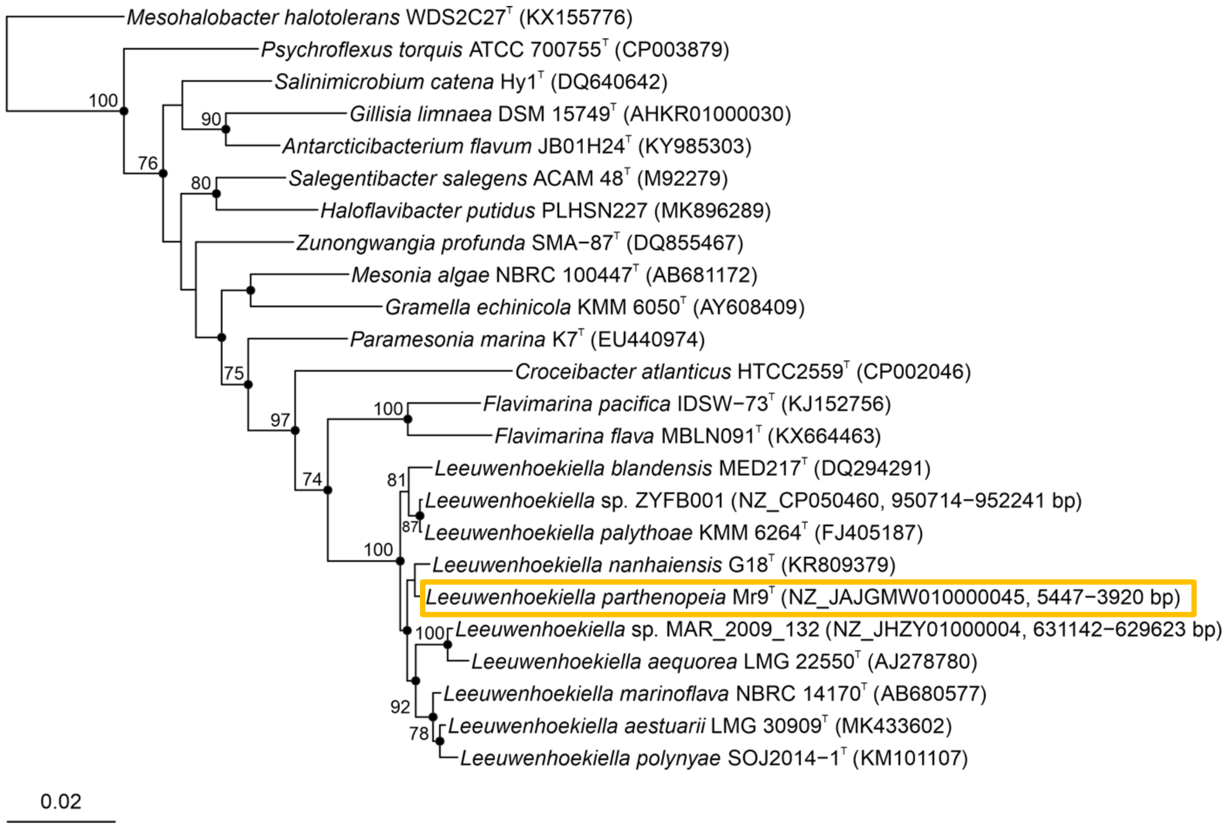

Supplementary Figure 2. Neighbor-joining phylogenetic tree based on the 16S rRNA gene sequence comparison of members of the genus *Leeuwenhoekiella*, obtained with matrix distance computed using the model of DNA evolution Jukes-Cantor. Bootstrap values based on 1000 *pseudo*-replicates ≧ 70% are shown. The filled circles indicate clusters recovered using the three algorithms, maximum-parsimony, maximum-likelihood, and neighbor-joining (shown). *Mesohalobacter halotolerans* WDS2C27^{T} was used as outgroup. Bar, 0.01 changes per nucleotide position.

PE = Phosphatidylethanolamine

AL = Aminolipid

GL = Glycolipid

L = Lipid

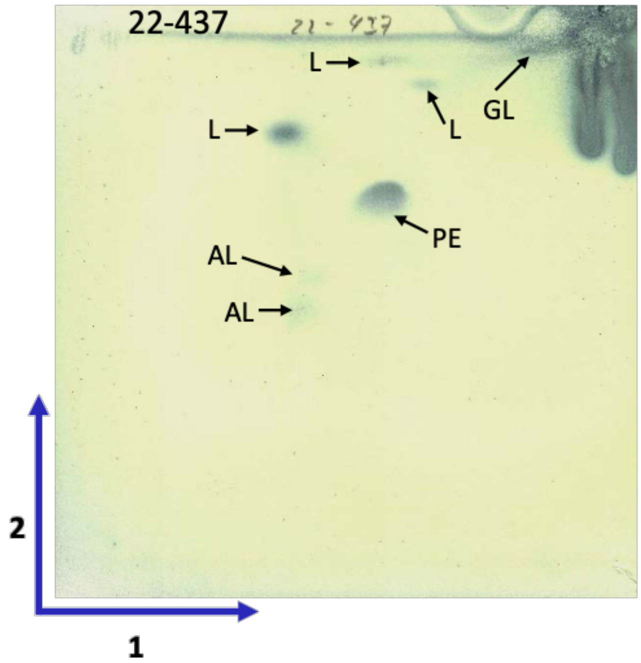

Supplementary Figure 3. Two-dimensional thin layer chromatography showing the polar lipid composition of *Leeuwenhoekiella parthenopeia* Mr9^T from deposited material (DSM 112950). Staining with phosphomolybdic acid.

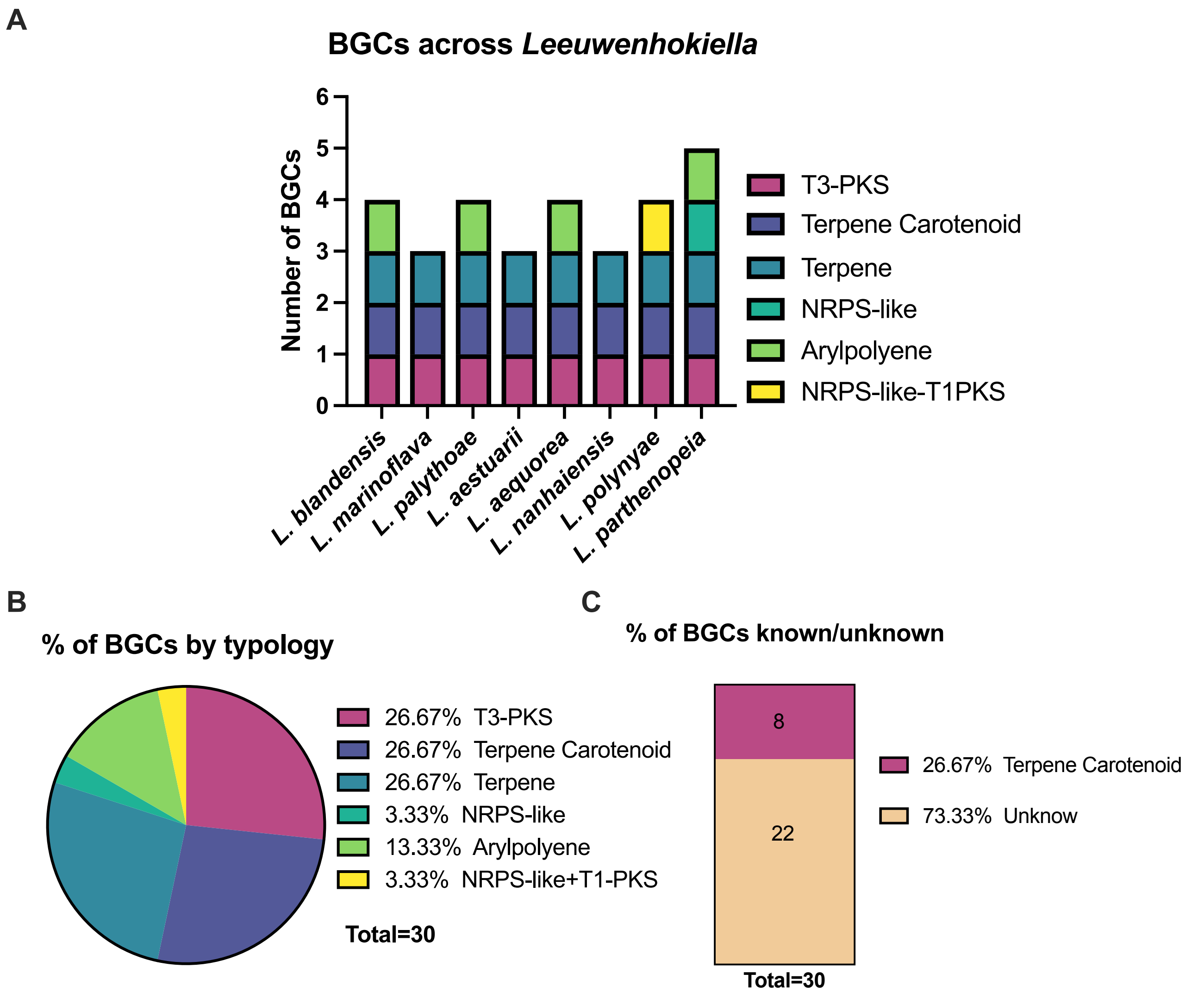

Supplementary Figure 4. (A) BGCs content of each species of *Leeuwenhoekiella.* **(B)** Percentage of BGCs by typology. **(C)** Ratio of percentage of BGCs with match with known BGCs encoding enzymes for the synthesis o known compounds.

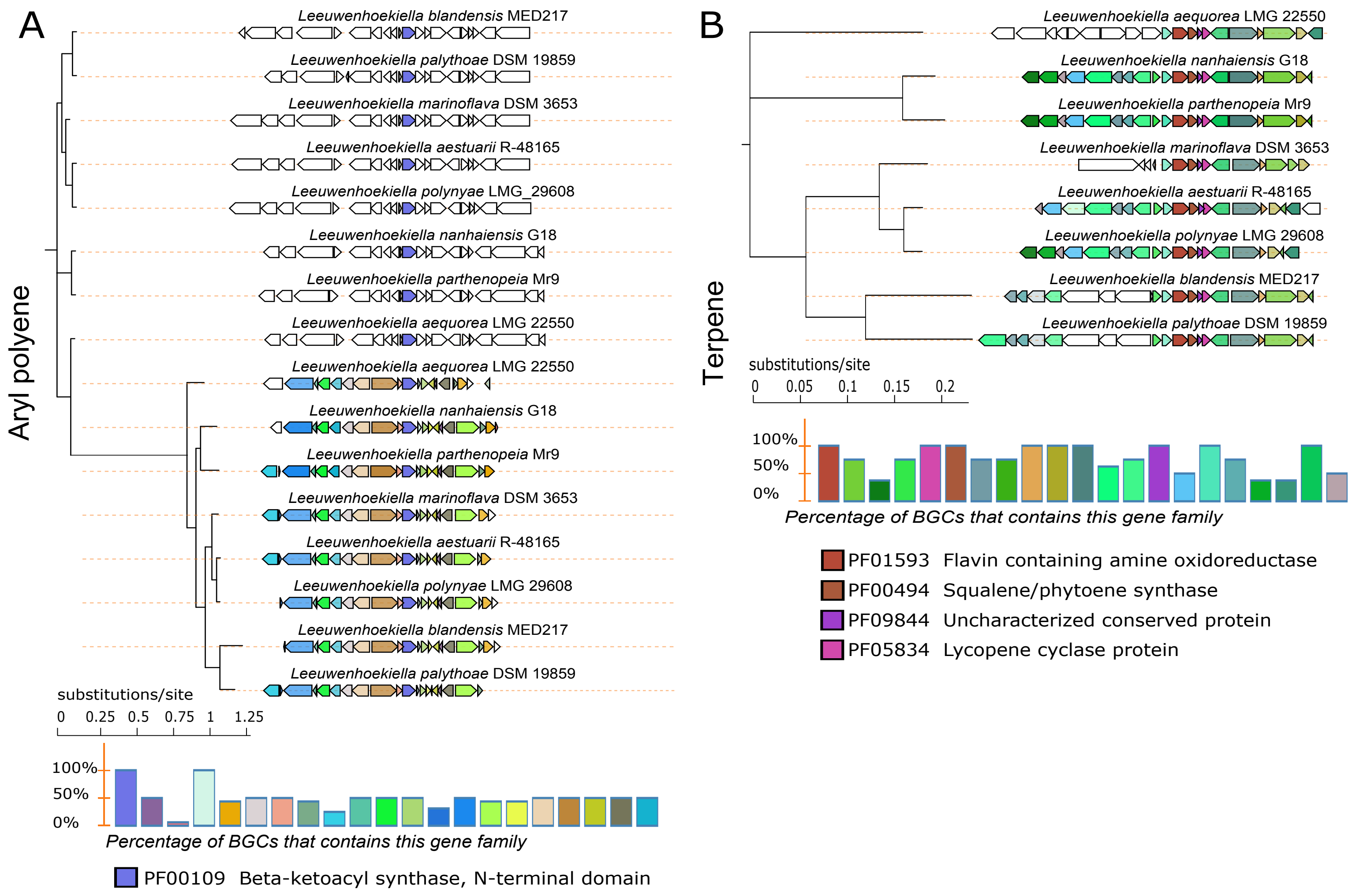

Supplementary Figure 5. Approximate-maximum-likelihood trees after phylogenetic reconstruction with CORASON based on the most conserved genes of the BGC family. **(A)** Superfamily tree of aryl polyene. **(B)** Superfamily tree of terpene.

Supplementary Table 1. Features of genome sequences of the genus *Leeuwenhoekiella* used in this study.

Species	***Strain***	***Accession No.***	***Assembly***	***Level***	***Size (Mb)***	***GC %***	***Scaffold***	***Contigs***	***CDS***	***N50***	***L50***
Leeuwenhoekiella palythoae	LMG 24856^T	QOVN00000000.1	GCF_004104045.1	contig	3.94	40.00	22	22	3326	619,136	3
Leeuwenhoekiella palythoae	DSM 19859^T	FQXT00000000.1	GCF_900130045.1	scaffold	4.03	39.90	14	17	3392	794,559	3
Leeuwenhoekiella sp.	ZYFB001^T	NZ_CP050460.1	GCF_011784865.1	Chromosome	4.05	40.00	1	1	3426	4,053,756	1
Leeuwenhoekiella blandensis	MED 217^T	AANC00000000.1	GCF_000152985.1	Chromosome	4.24	39.80	1	15	3735	392,634	4
Leeuwenhoekiella marinoflava	LMG 1345^T	QOVL00000000.1	GCF_004104035.1	contig	4.75	37.50	53	53	4065	190,456	8
Leeuwenhoekiella marinoflava	DSM 3653^T	FQUN00000000.1	GCF_900129005.1	contig	4.73	37.50	44	49	4056	194,792	8
Leeuwenhoekiella polynyae	LMG 29608^T	QOVK00000000.1	GCF_004104075.1	contig	4.79	38.1	47	47	4075	173,556	7
Leeuwenhoekiella aestuarii	R-48165^T	QOVJ00000000.1	GCF_004104055.1	contig	4.43	37.80	30	30	3085	637,981	3
Leeuwenhoekiella nanhaiensis	G18^T	NQXA00000000.1	GCF_002744755.1	scaffold	4.37	42.1	50	60	3768	262,916	4
Leeuwenhoekiella parthenopeia	**Mr9^T**	**JAJGMW000000000.1**	**GCF_020782115.1**	**contig**	**4.42**	**42.20**	**58**	**58**	**3700**	**146,356**	**10**
Leeuwenhoekiella aequorea	LMG 22550^T	QOVM00000000.1	GCF_004104375.1	contig	3.46	35.4	18	18	3102	501,718	3
Leeuwenhoekiella sp.	MAR 2009 132^T	JHZY00000000.1	GCF_000687915.1	contig	4.28	37.00	5	5	3608	2,318,059	1

Supplementary Table 2. Metagenomic dataset used in this study.

Country/Date/depth	Metagenome	BioProject	Sequence read archive (SRA)
Algeria-SEP2009-42m	TARA_007_42m	PRJEB1788	ERR315856
Algeria-SEP2009-5m	TARA_007_05m	PRJEB1788	ERR315857
Croatia-NOV2009-5m	TARA_023_05m	PRJEB1787	ERR315858
Croatia-NOV2009-55m	TARA_023_55m	PRJEB1787	ERR315859
Cyprus-DIC2009-5m	TARA_030_05m	PRJEB1787	ERR315862
Cyprus-DIC2009-70m	TARA_030_70m	PRJEB1787	ERR318618
West-Sardinia-SEP2009-55m	TARA_009_55m	PRJEB1788	ERR594315
West-Sardinia-SEP2009-5m	TARA_009_05m	PRJEB1788	ERR594317
Greece-NOV2009-5m	TARA_025_05m	PRJEB1787	ERR598951
Greece-NOV2009-50m	TARA_025_50m	PRJEB1787	ERR599094
Malta-NOV2009-5m	TARA_018_05m	PRJEB1787	ERR598993
Malta-NOV2009-60m	TARA_018_60m	PRJEB1787	ERR599073
AegeanSea-OCT2010-75m	Med-Ae1-75mDCM	PRJNA305355	SRR2981519
AegeanSea-OCT2010-600m	Med-Ae2-600mDeep	PRJNA305355	SRR2981523
Spain-JUL2012-75m	MedDCM-JUL2012-75mDCM	PRJNA257723	SRR1539383
Spain-SEP2013-55m	MedDCM-SEP2013-55m	PRJNA257723	SRR1539645
Spain-SEP2015-60m	MedDCM-SEP2015-60m	PRJNA257723	SRR5010551
IonianSea-OCT2010-70m	Med-Io16-70mDCM	PRJNA305355	SRR2981512
IonianSea-OCT2010-3000m	Med-Io17-3000mDeep	PRJNA305355	SRR2981515
IonianSea-OCT2010-77m	Med-Io7-77mDCM	PRJNA305355	SRR2981506
Spain-SEP2014-15m	Med-SEP2014-15m	PRJNA257723	SRR5877534
Spain-SEP2014-30m	Med-SEP2014-30m	PRJNA257723	SRR5877535
Spain-SEP2014-60m	Med-SEP2014-60m	PRJNA257723	SRR3993157
Spain-OCT2015-15m	Med-OCT2015-15m	PRJNA352798	SRR5007106
Spain-OCT2015-30m	Med-OCT2015-30m	PRJNA352798	SRR5007114
Spain-OCT2015-45m	Med-OCT2015-45m	PRJNA352798	SRR5007115
Spain-OCT2015-60m	Med-OCT2015-60m	PRJNA352798	SRR5007118
Spain-OCT2015-75m	Med-OCT2015-75m	PRJNA352798	SRR5007138
Spain-OCT2015-90m	Med-OCT2015-90m	PRJNA352798	SRR5007139
Spain-OCT2015-1000m	Med-OCT2015-1000m	PRJNA352798	SRR5007141
Spain-OCT2015-2000m	Med-OCT2015-2000m	PRJNA352798	SRR5007147
Spain-OCT2015-15m-7h	Med-OCT2015-15m-7h	PRJNA352798	SRR8503606
Spain-OCT2015-15m-14h	Med-OCT2015-15m-14h	PRJNA352798	SRR8503605
Spain-OCT2015-60m-7h	Med-OCT2015-60m-7h	PRJNA352798	SRR8503608
Spain-OCT2015-60m-14h	Med-OCT2015-60m-14h	PRJNA352798	SRR8503607
Spain-OCT2015-90m-7h	Med-OCT2015-90m-7h	PRJNA352798	SRR8503604
Spain-DEC2013-20m	MedWinter-DEC2013-20m	PRJNA257723	SRR5877432
Spain-JAN2015-20m	MedWinter-JAN2015-20m	PRJNA257723	SRR3405540
Spain-JAN2015-80m	MedWinter-JAN2015-80m	PRJNA257723	SRR5877433
Spain-Feb2019-20m	MedWinter-Feb2019-20m	PRJNA674982	SRR13009789
Spain-Feb2019-40m	MedWinter-Feb2019-40m	PRJNA674982	SRR13009789

Supplementary Table 3. BGC's account of the genus *Leeuwenhoekiella* showing homologies with known BGC's present in other organisms.

Name	***Genome Size bp***	***Nº BGC's***	***Type***	***Length BGC***	***Most similar know cluster (compound)***	***Similarity***	***Organism***
L. blandensis							
			terpene	21,155		-	
	4,245	4	T3PKS	41,068		-	
			terpene	20,840	carotenoid terpene	28%	*Myxococcus xanthus*
			Aryl polyene	41,269		-	
L. marinoflava							
			terpene	19,936		-	
	4,733	3	T3PKS	41,092		-	
			terpene	20,840	carotenoid terpene	28%	*Myxococcus xanthus*
L. palythoae							
			terpene	21,149		-	
			terpene	20,840	carotenoid terpene	28%	*Myxococcus xanthus*
	4,030	4	Aryl polyene	41,269		-	
			T3PKS	41,092		-	
L. aestuarii							
			terpene	21,149		-	
	4,430	3	terpene	20,840	carotenoid terpene	28%	*Myxococcus xanthus*
			T3PKS	31.745		-	
L. aequorea							
			terpene	21,149		-	
	3,458	4	Aryl polyene	41,269		-	
			T3PKS	41,068		-	
			terpene	20,840	carotenoid terpene	28%	*Myxococcus xanthus*
L. nanhaiensis							
			terpene	21,149		-	
	4,370	3	terpene	20,84	carotenoid terpene	28%	*Myxococcus xanthus*
			T3PKS	41.077		-	
L. polynyae							
			T3PKS	41,092		-	
	4,794	4	terpene	20,840	carotenoid terpene	28%	*Myxococcus xanthus*
			terpene	21,149			
			NRPS-like - T1PKS	38,218		-	
L. parthenopeia							
			terpene	20,840	carotenoid terpene	28%	*Myxococcus xanthus*
			terpene	21,149		-	
	4,419	5	Aryl polyene	41,275		-	
			T3PKS	38,296		-	
			NRPS-like	34,825			
